# Cochlear Implantation From the Perspective of Genetic Background

**DOI:** 10.1002/ar.24360

**Published:** 2020-02-06

**Authors:** Shin‐ichi Usami, Shin‐ya Nishio, Hideaki Moteki, Maiko Miyagawa, Hidekane Yoshimura

**Affiliations:** ^1^ Department of Otorhinolaryngology Shinshu University School of Medicine Matsumoto Japan; ^2^ Department of Hearing Implant Sciences Shinshu University School of Medicine Matsumoto Japan

**Keywords:** cochlear implant, hearing loss, gene, genetic etiology, electric acoustic stimulation

## Abstract

While cochlear implantation (CI) technology has greatly improved over the past 40 years, one aspect of CI that continues to pose difficulties is the variability of outcomes due to numerous factors involved in postimplantation performance. The electric acoustic stimulation (EAS) system has expanded indications for CI to include patients with residual hearing, and is currently becoming a standard therapy for these patients. Genetic disorders are known to be the most common cause of congenital/early‐onset sensorineural hearing loss, and are also involved in a considerable proportion of cases of late‐onset hearing loss. There has been a great deal of progress in the identification of deafness genes over the last two decades. Currently, more than 100 genes have been reported to be associated with non‐syndromic hearing loss. Patients possessing a variety of deafness gene mutations have achieved satisfactory auditory performance after CI/EAS, suggesting that identification of the genetic background facilitates prediction of post‐CI/EAS performance. When the intra‐cochlear etiology is associated with a specific genetic background, there is a potential for good CI performance. Thus, it is essential to determine which region of the cochlea is affected by identifying the responsible genes. This review summarizes the genetic background of the patients receiving CI/EAS, and introduces detailed clinical data and CI/EAS outcomes in representative examples. Anat Rec, 303:563–593, 2020. © 2020 The Authors. *The Anatomical Record* published by Wiley Periodicals, Inc. on behalf of American Association of Anatomists.

## GENERAL RELATIONSHIP BETWEEN CI OUTCOME AND THE GENETIC BACKGROUND OF PATIENTS

### Cochlear Implantation and Deafness Genes

**Figure 1:**
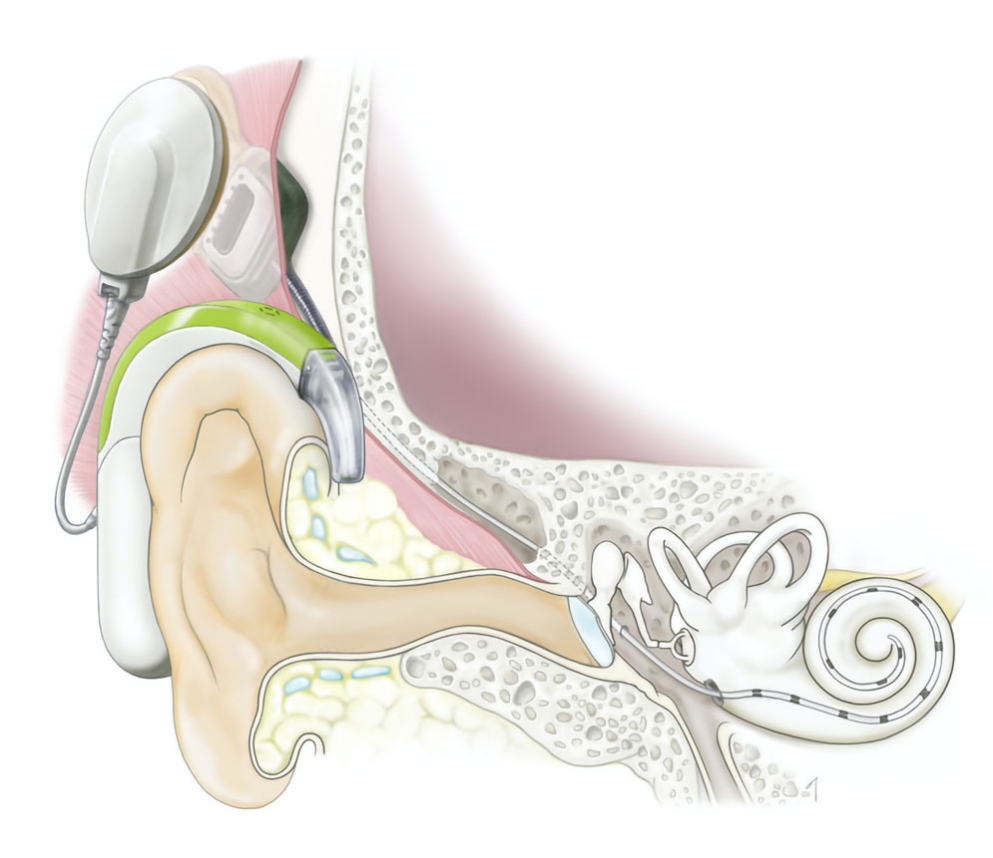
Schematic image of cochlear implantation. The cochlear implant consists of two parts; an audio processor and implant. The audio processor contains microphones, digital signal processor chips, a battery, and an antenna which sends electric stimulation to the implant device which is surgically implanted. The coil of the implant receives the electric signal from the audio processor and sends electric stimulation to the cochlear nerve *via* the electrode inserted into the scala tympani of the cochlea.

Cochlear implantation (CI) is the surgical insertion of a device to provide electrical stimulation directly to the auditory nerve (Fig. 1). CI is able to bypass the cochlea in which the pathogenetic cause lies. CI is currently the standard therapeutic option for severe‐to‐profound sensorineural hearing loss patients.

**Figure 2:**
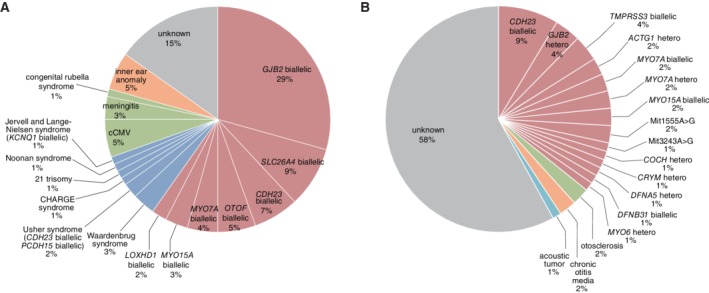
Genetic background of the CI patients. Genetic testing successfully identified causative mutations in deafness genes in A: ~60% of prelingual hearing loss patients, and B: about 36% of postlingual hearing loss patients (Miyagawa et al., 2016).

Although CI provides a good outcome for the majority of cases, factors affecting the outcomes of CI vary among patients. One reason of such variations is thought to be the heterogeneous cause of hearing loss. The function of the cochlea could be affected by various factors, including genetic factors, viral infections, and congenital anomalies. Among them, genetic factors represent the most common etiology in severe‐to‐profound hearing loss, and might be one of the key determinants of outcomes for CI and electric acoustic stimulation (EAS) (Miyagawa et al., 2016) (Fig. 2). Genetic testing has successfully identified the causative mutations in 60% of patients with prelingual onset hearing loss and in 36% of those with postlingual hearing loss (Fig. 2). With regard to the responsible gene, the most frequent causative gene was *GJB2* (29%), followed by *SLC26A4* (9%), *CDH23* (7%), *MYO7A* (4%), *OTOF* (5%), *MYO15A* (3%), and *LOXHD1* (2%), indicating that these deafness genes are typical deafness genes found in the prelingual CI/EAS patients. A further 9% of the patients were diagnosed with syndromic deafness associated with other symptoms. For postlingual CI/EAS patients, a genetic etiology was detectable in ~36% of cases. Although genetic causes were the most common, a number of different kinds of causative genes, including various rare genes, were found to be involved in postlingual CI/EAS patients. The most common causative gene was *CDH23* (9%), followed by *MYO7A* (4%), *TMPRSS3* (4%), *MYO15A* (2%), *DFNB31* (1%), *ACTG1* (2%), *DFNA5* (1%), *MYO6* (1%), and *CRYM* (1%). In the postlingual CI/EAS patients, mitochondrial m.3243A>G (1%) and m.1555A>G mutations (2%) were also found to be involved. Compared to the prelingual group, many genes with autosomal dominant inheritance, such as *MYO7A*, *ACTG1*, *DFNA5*, *MYO6*, and *CRYM*, as well as mitochondrial genes reported to cause progressive hearing loss, were found to be involved.

### Screening Strategy for Deafness Genes

**Figure 3:**
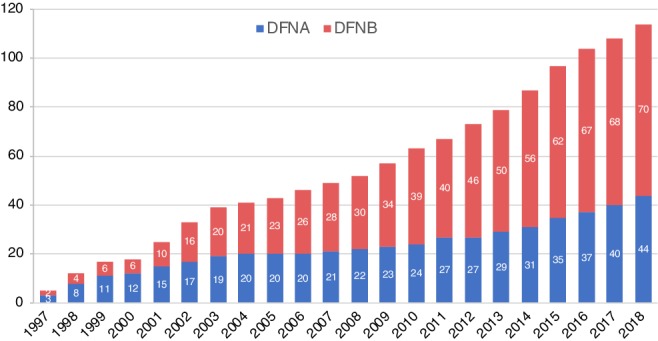
Total numbers of deafness genes for non‐syndromic hearing loss reported in the literature. Blue: autosomal dominant inherited hearing loss causative genes (DFNA), red: autosomal recessive inherited hearing loss causative genes (DFNB) calculated based on the Hereditary Hearing Loss Homepage (Van Camp G and Smith RJH. Hereditary Hearing Loss Homepage. http://hereditaryhearingloss.org).

In the past two decades, there has been considerable progress in the identification of deafness genes. Currently, more than 100 genes have been reported for non‐syndromic hearing loss (Fig. 3). Due to the extreme heterogeneity of the disorder, precise diagnosis of hereditary hearing impairment can be difficult. One‐by‐one conventional screening based on Sanger sequencing is an accurate and reliable method, but it is also time‐consuming and has limited application to comprehensive screening programs. Toward a more comprehensive diagnosis to covering more causative genes, we are now applying Massively Parallel DNA Sequencing using a next‐generation sequencer. Genetic analysis using this technology has allowed the identification of rare mutations in relatively uncommon causative genes, thereby improving the diagnostic rate (Nishio and Usami, 2015).

### Genetic Epidemiology

**Figure 4:**
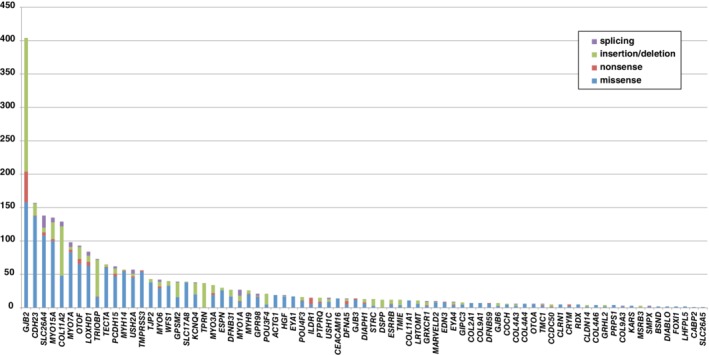
Genetic epidemiology based on comprehensive NGS analysis results. *GJB2* is the most prevalent causative gene, and the major (commonly found) gene mutations in GJB2 cause deafness in 30%–40% of cases. The remaining cases of hearing loss are the result of various rare genes/mutations that have been difficult to diagnose by the conventional one‐by‐one approach (Nishio and Usami, 2015).

Recent comprehensive next‐generation sequencing (NGS) analysis has helped clarify genetic epidemiology; i.e., *GJB2* is the predominant deafness‐causing gene, with the other common genes being *CDH23*, *SLC26A4*, *MYO15A*, *COL11A2*, and *MYO7A* (Miyagawa et al., 2013; Nishio and Usami, 2015) (Fig. 4). The remaining cases of hearing loss arose from various rare genes/mutations that were not easy to identify using the conventional one‐by‐one screening approach. These epidemiological data indicate that there are significant differences in the responsible genes between the congenital group and late‐onset group (Miyagawa et al., 2013).

### Molecules Encoded by Deafness Genes

The cochlea is composed of various types of cells. The coordinated action of various molecules is essential for the normal development and maintenance of auditory processing in the cochlea. A series of studies have clarified the localization of these molecules in the cochlea (Nishio et al., 2015 for review).

**Figure 5:**
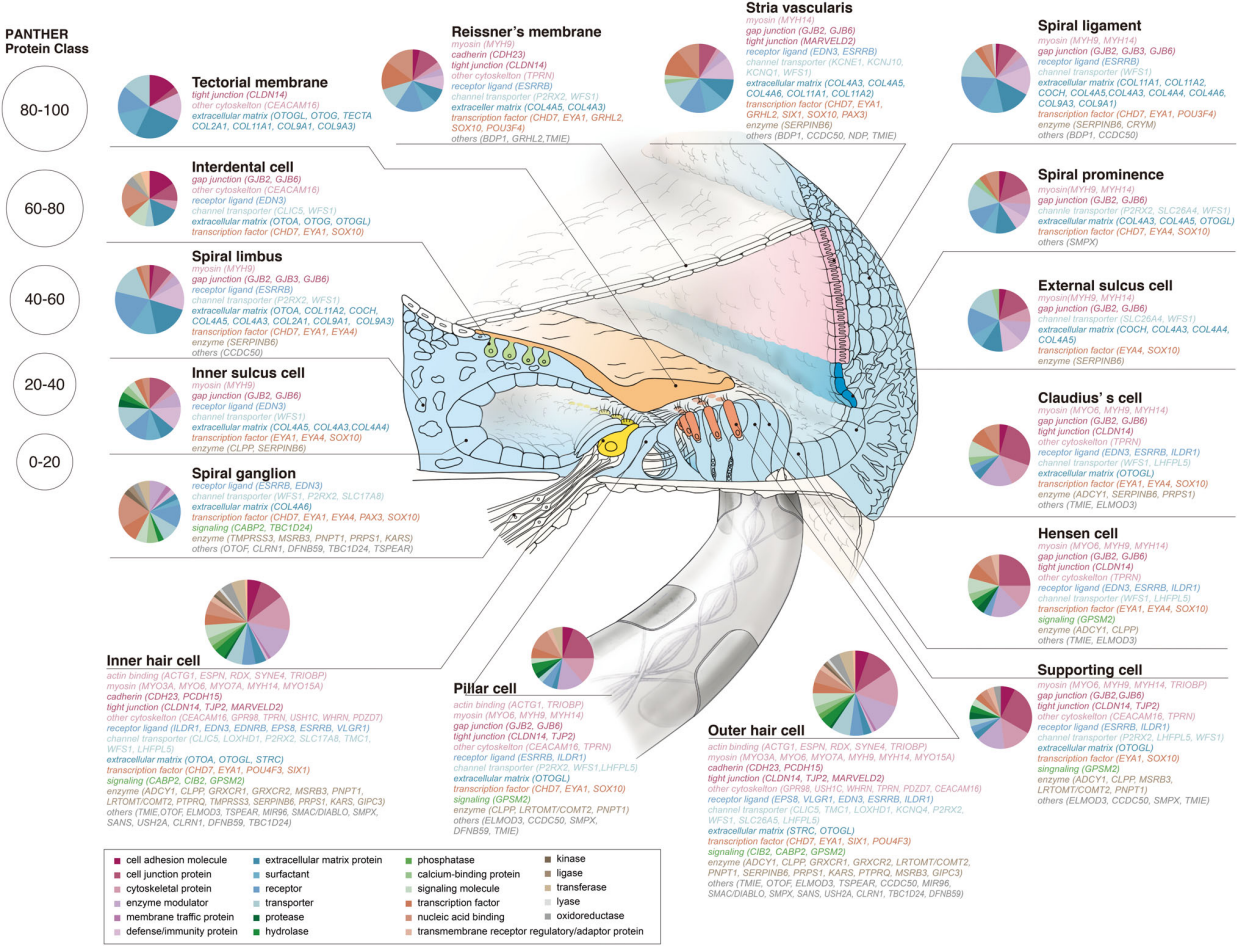
Gene expression profiles of the causative genes and localization of the encoded proteins involved in hereditary hearing loss in the cochlea. Pie charts indicate the results of gene ontology analysis of the gene expression profiles for each cell type (modified from Nishio et al., 2015).

The key molecules encoded by deafness genes have been studied from both the morphological and physiological viewpoints, and a series of *in situ* hybridization and immunohistochemical studies have clarified the precise localization of these molecules in the cochlea and vestibular end organs (Fig. 5).

In terms of clinical applications, the most remarkable aspect of these advances is that ENT clinicians can now make highly accurate molecular diagnoses through the use of genetic testing, enabling a clearer understanding of the mechanisms involved, more appropriate and precise treatment selection, and greatly improved genetic counseling.

### Gene Expression Patterns

Gene expression analysis using a laser capture micro‐dissection technique revealed gene expression in individual regions. Each portion (spiral ganglion, organ of Corti, spiral limbus, lateral wall) was separated, and then total RNA was extracted from each slice and converted to cDNA. Each gene has a specific expression pattern (Nishio et al., 2017). In this review, gene expression patterns are shown in the figures to provide a better understanding of the relationship between gene expression and CI outcome. However, some genes are found to have extremely limited expression; therefore, it is difficult to discuss their correlation with CI outcomes.

### Etiology and CI Outcome

**Figure 6:**
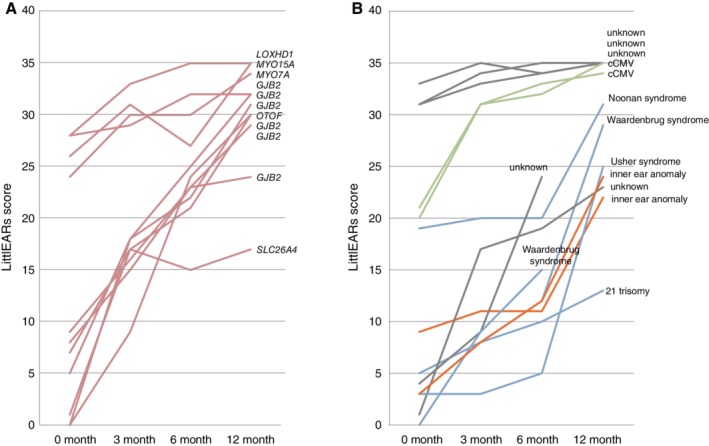
Early auditory development assessed after cochlear implantation using the LittlEARS auditory questionnaire. **A**: Non‐syndromic hearing loss with specific gene mutations (n = 11). **B**: Other etiology (n = 11). Pink: non‐syndromic hearing loss associated with specific gene mutations. Blue: syndromic hearing loss. Green: infection‐induced hearing loss. Orange: inner ear anomalies. Gray: unknown (Miyagawa et al., 2016).

When the cause of deafness is involved in the “intra‐cochlear” etiology (associated with mutations in a number of deafness genes known to be expressed inside the cochlea), good CI outcomes may potentially be achieved. Therefore, efforts for the identification of the region of the cochlea involved (either inside or outside the cochlea) should be made by delineating the responsible genes. Regarding the outcomes of CI, the LittlEARS auditory questionnaire was used to assess the auditory behavior development before the operation and at 3, 6, and 12 months after CI. Although scores varied among the patients, the majority of non‐syndromic hearing loss patients with specific deafness gene mutations showed good and rapid development of auditory behavior. In contrast, syndromic hearing loss patients as well as the patients with inner ear anomalies showed comparatively poorer and slower development (Miyagawa et al., 2016) (Fig. 6).

For children with congenital severe‐to‐profound hearing loss, simultaneous bilateral CI is becoming popular in many CI centers. Genetic diagnosis is important for decision making. If deaf children have genetic mutations in a gene specifically expressed in the inner ear, simultaneous bilateral CI is strongly recommended as the intra‐cochlear etiology might be a prognostic factor for favorable CI outcomes.

For postlingual patients, evaluation was rather difficult because the etiology is thought to be more complex, but a similar tendency was found in our cohort (Miyagawa et al., 2016). When hearing loss is associated with mutations in genes expressed within the cochlea, word recognition scores are usually dramatically improved after CI. Among the multiple factors, in addition to age, we found differences in etiology; i.e., intra‐cochlear etiology was detected in 40%–43% of the good CI outcome patients whereas only in 23%–27% of the poorer CI outcome group.

**Figure 7:**
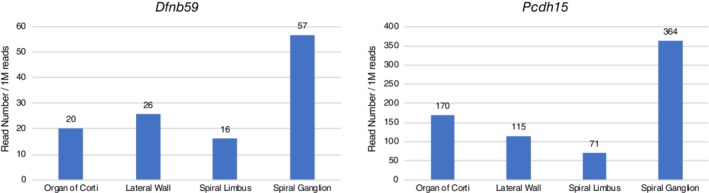
Gene expression profiles for the *Dfnb59* and *Pcdh15* genes. The gene expression analysis results of a laser micro‐dissection‐based gene expression study which showed that both *Dfnb59* and *Pcdh15* are highly expressed outside the cochlea (Nishio et al., 2017).

Genes associated with poor CI outcomes have also been reported. The patients with *DFNB59* or *PCDH15* variants are associated with poor CI performance (Wu et al., 2015). This is in line with the gene expression results of a laser‐microdissection‐based gene expression study which showed that *DFNB59* and *PCDH15* are highly expressed outside the cochlea (Nishio and Usami, 2017) (Fig. 7).

### Progress of CI

**Figure 8:**
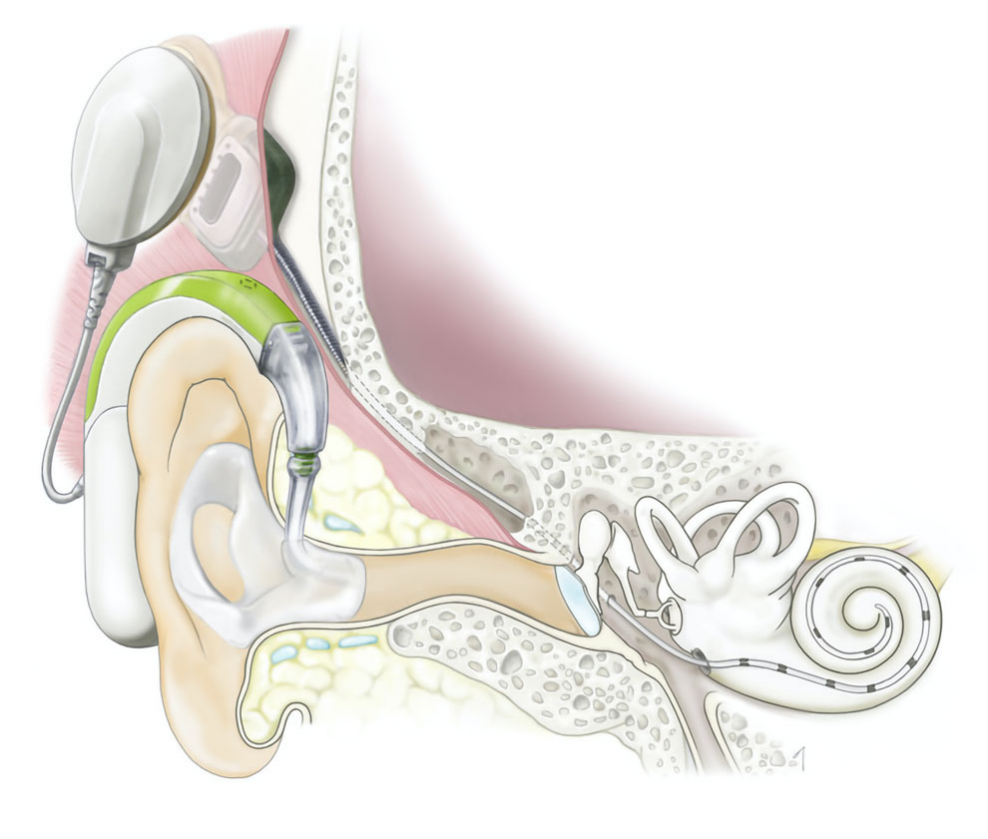
Schematic image of electric acoustic stimulation. EAS uses a combination of acoustic stimulation via the external auditory canal for lower frequency sound and electric stimulation via CI for higher frequency sound.

There has been a great deal of progress in cochlear implant science in these past two decades. The indication for CI was originally for the patients with profound hearing loss in all frequencies, but EAS has expanded indications for CI to include patients with residual hearing. EAS, which uses a combination of acoustic stimulation via the external auditory canal and electric stimulation via CI, was first introduced in 1999 (Fig. 8, see von Ilberg et al., 2011 for review) For those patients retaining lower frequency residual hearing, EAS is currently becoming a standard therapy.

### Importance of Genetic Testing for EAS Patients

Various genetic etiologies are known to be involved in patients receiving EAS, with the majority of the patients showing favorable outcomes (Usami et al., 2012; Miyagawa et al., 2013). However, selection of the appropriate electrode and prediction of outcomes can be difficult as progression of hearing loss varies depending on individual differences, being sometimes fairly rapid and sometimes relatively stable. Onset age as well as progression speed of hearing loss appear to vary according to the etiology; therefore, identification of the responsible genes may be helpful in decisions for EAS/CI surgery and in the selection of the appropriate device and/or electrode.

In addition, there are considerable numbers of children with residual hearing; accordingly, the evaluation of residual hearing and estimation of progression is important. However, for very young children, it is difficult to evaluate residual hearing in the low frequencies. Genetic testing is advantageous in that we can predict the possible prognosis for hearing; i.e., whether it is progressive or not, for individual patients.

We have published a series of papers concerning the genetic background of patients with CI/EAS. This review summarizes the responsible genes reported in CI patients and discusses what we have learned from the progress of two newly progressing fields of science, CI and the genetic background of patients. Detailed clinical data and outcomes of CI are shown in representative examples we personally evaluated.

## CI IN PATIENTS WITH SPECIFIC GENETIC BACKGROUNDS

### *GJB2*

**Figure 9:**
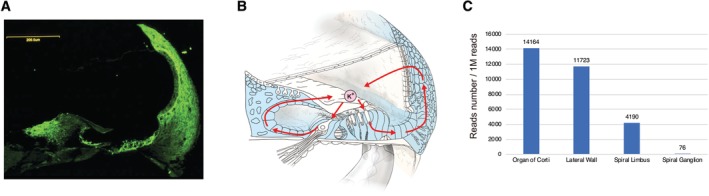
Gene expression profiles for the *GJB2* gene. **A**: Immunohistochemical localization of the connexin 26 protein in the rat cochlea. **B**: A scheme of potassium recycling in the cochlea. The pale blue region indicates the cells expressing *Gjb2*. **C**: *Gjb2* gene expression in the mouse cochlea and spiral ganglion (modified from Nishio et al., 2017).

*GJB2*, which codes a gap junction protein, connexin 26 (Cx26), was the first gene identified as being involved with non‐syndromic hearing impairment (Kelsell et al., 1997). Cx26 is distributed in the spiral ligament, basal cells of the stria vascularis, various supporting cells, and limbal fibrocytes, and is considered to play a major role in intracellular communication as well as in the recycling of potassium ions (Kikuchi et al., 1995) (Fig. 9).

*GJB2* is the most prevalent gene known to be responsible for congenital hearing loss worldwide, and consequently is the focus of universal newborn hearing screening programs. Approximately 25% of congenital subjects possess at least one *GJB2* mutation (Tsukada et al., 2010), and our previous results indicated that *GJB2* is also the predominant causative gene among prelingual CI patients (Miyagawa et al., 2016). To date, more than 100 *GJB2* variants have been reported (see the Connexin‐deafness homepage: http://davinci.crg.es/deafness/), and there has been a general rule applied to the relationship between mutations and hearing loss; that inactivating mutations (deletion mutations and stop mutations) result in more severe phenotypes compared to those caused by non‐inactivating mutations (missense mutations) (Tsukada et al., 2010). As well as allowing highly accurate diagnoses, these genotype–phenotype correlation data can provide prognostic information to help decide the intervention strategy for patients with hearing loss; i.e., whether a child should receive CI or hearing aids. For patients with severe phenotypes who possess *GJB2* mutations, genetic information would aid in decision making regarding CI, as their hearing loss is of cochlear origin and they, therefore, would be good candidates for CI. The present literature review indicates that the majority of papers showed significant improvement in speech performance in *GJB2*‐related deafness patients and the remaining papers showed equivalent results between *GJB2‐*related deafness and non‐*GJB2* deafness (for review; Abdurehim et al., 2017; Nishio and Usami, 2017). The difference in these results is probably due to patient selection bias. It should be noted that no literature has reported poorer outcomes for CI in patients with *GJB2* mutations.

### Case: Congenital Hearing Loss Indicative for CI

**Figure 10:**
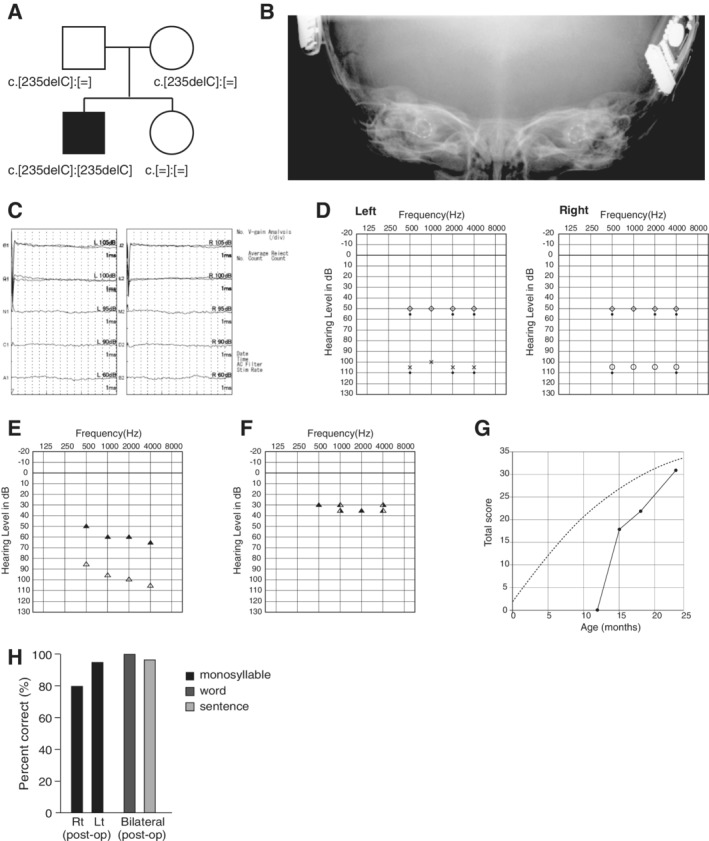
**A**: Pedigree of a patient with *GJB2* mutations. **B**: Postoperative X‐ray findings. **C**: ABR findings at three months old showing profound hearing loss. **D**: ASSR findings at three months old showing hearing loss in all frequencies and no residual hearing in the lower frequencies. **E**: Hearing amplification with hearing aids indicating insufficient amplification to obtain good language development. **F**: Hearing thresholds after bilateral CI. **G**: Auditory behavioral development assessed by LittlEARS auditory questionnaire. The development curve shows rapid improvement in auditory behavior, reaching the curve of normally developed children. **H**. Speech perception test results at Age 7 were dramatically improved (monosyllable: rt 80%, lt 95%; bil word: 100%; bil sentence: 96%).

The patient had homozygous *GJB2* mutations (c.[235delC]; [235delC]), and the parents were found to be carriers for the mutation (Fig. 10A). His hearing was screened by otoacoustic emission (OAE) during newborn screening, and he was found to have hearing loss. At the age of three months, he was evaluated by auditory steady state response (ASSR) and auditory brainstem response (ABR), showing profound hearing loss in all frequencies (Fig. 10C,D). Conditioned orientation reflex (COR) evaluated at eight months of age showed insufficient amplification to obtain good language development (Fig. 10E) and he received a left CI (MED‐EL PULSAR CI100/standard electrode) at 10 months (Fig. 10B). Auditory development was observed after nine months of CI use: Infant‐Toddler Meaningful Auditory Integration Scale (IT‐MAIS) 16/40>25/40, LittlEARS 28>33 (Fig. 10G). Speech perception test results at age 7 were dramatically improved (monosyllable: rt 80%, lt 95%; word: 100%; sentence: 96%) (Fig. 10H) and the patient currently goes to a regular school. As in a series of literature, this case demonstrated that CI has brought about tremendous improvements in auditory skills as well as in speech production development in patients with profound hearing loss resulting from *GJB2* mutations.

### *SLC26A4*

**Figure 11:**
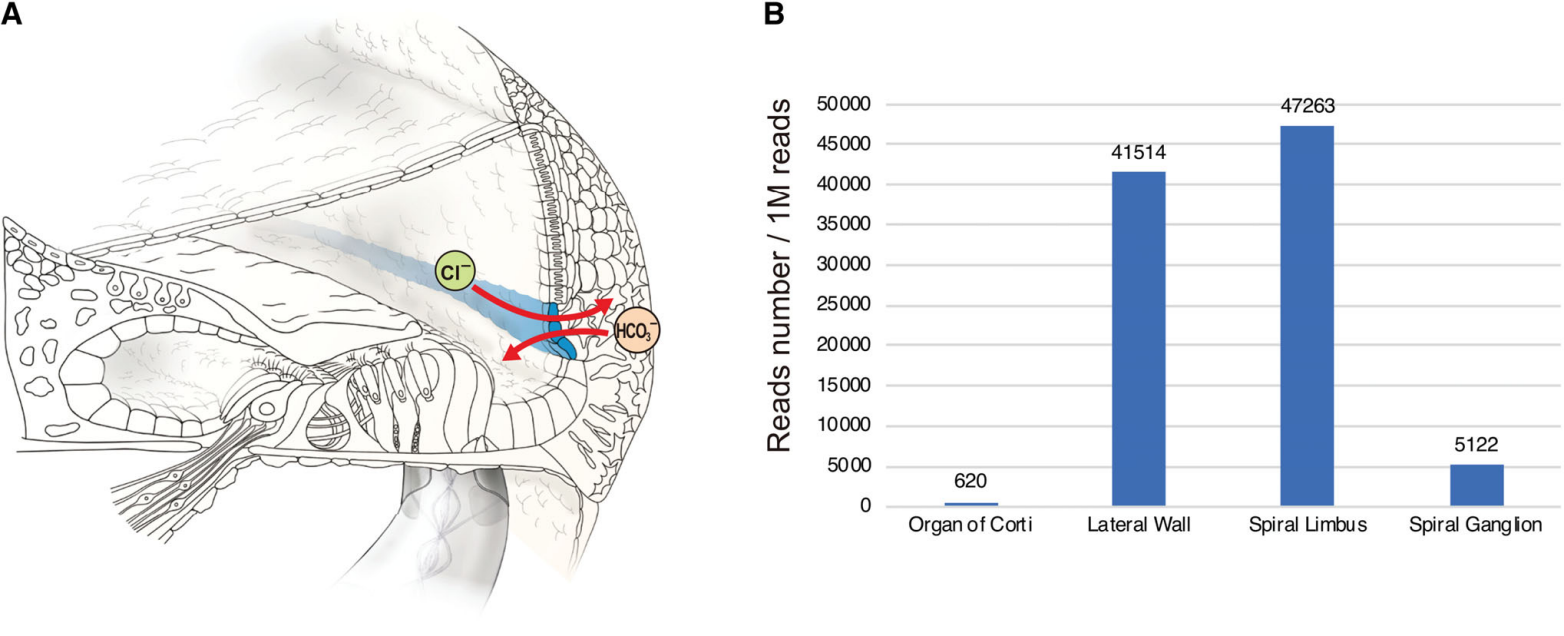
**A**: Immunocytochemical localization of pendrin in the cochlea. Pendrin is expressed in the spiral prominence of the cochlea and acts as a transporter for chloride ions and bicarbonate ions. **B**: *Slc26a4* gene expression in the mouse cochlea and spiral ganglion (modified from Nishio et al., 2017).

**Figure 12:**
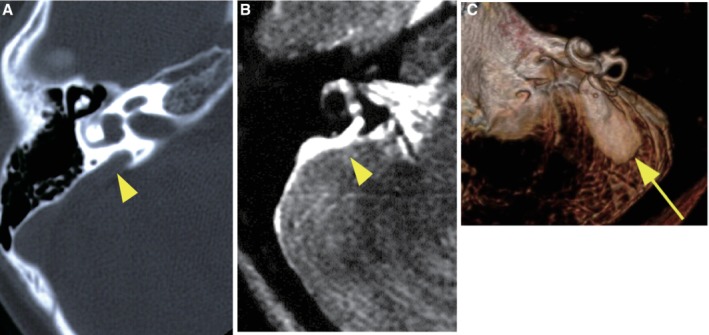
**A**: Computed tomography (CT) scan of an enlarged vestibular aqueduct (arrowhead), **B**: Magnetic resonance (MR) image of an enlarged endolymphatic duct (arrowhead), **C**: 3D imaging of an MR image of an enlarged endolymphatic duct and sac (arrow).

**Figure 13:**
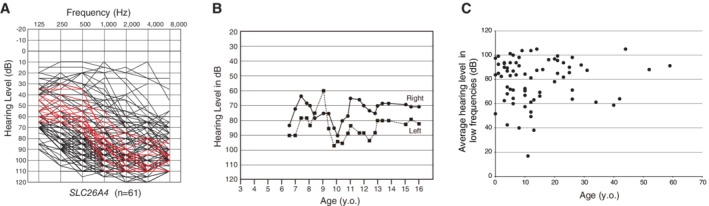
**A**: Overlapping audiograms of patients with biallelic *SLC26A4* mutations, showing high‐frequency involved sensorineural hearing loss with residual hearing at the lower frequencies (Usami et al., 2010), **B**: The hearing level of a *SLC26A4*‐associated hearing loss patient fluctuates (Usami et al., 1999), **C**: The hearing loss of the *SLC26A4*‐associated hearing loss patient is progressive (Miyagawa et al., 2014).

The *SLC26A4* gene is known to be expressed abundantly in the inner ear and thyroid (Everett et al., 1997). The encoded protein, pendrin, is localized in the outer sulcus cells and participates in regulating volume homeostasis through its ability to function as a chloride‐formate exchanger (Kim and Wangemann, 2010) (Fig. 11). Mutations in the *SLC26A4* gene are known to be responsible for a wide phenotypic spectrum, ranging from Pendred syndrome (a disorder associated with sensorineural hearing loss and thyroid goiters) to non‐syndromic hearing loss with enlarged vestibular aqueducts (EVA, Fig. 12) (DFNB4) (Miyagawa et al., 2014). High‐frequency involved, fluctuating, and progressive hearing loss are characteristic features of patients with EVA (Fig. 13). Hearing usually remains in the low frequencies; therefore, patients can understand spoken language with the use of hearing aids. However, a considerable number of patients experience progressive deterioration in the degree of hearing loss and, therefore, become candidates for EAS or CI (Fig. 13A). As with *GJB2* mutations, CI has enabled remarkable improvements in auditory skill as well as in speech perception in those with profound hearing loss associated with *SLC26A4*. Therefore, genetic information is important for predicting the outcome of CI and, therefore, decision making with regard to the mode of intervention.

### Case: Congenital Hearing Loss Indicative for CI

**Figure 14:**
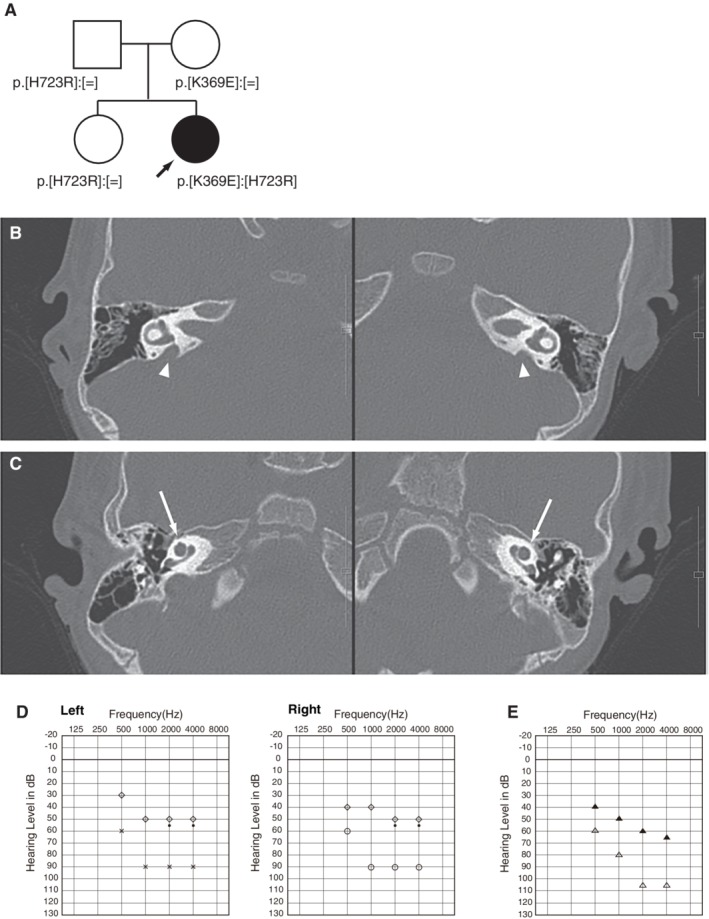
**A**: Pedigree of the patient with *SLC26A4* mutations. **B**: CT scans showing bilateral EVA (arrow heads) with **C**: cochlear hypoplasia (arrows). **D**: ASSR and **E**: COR findings. ASSR findings at six months old showing profound hearing loss with some residual hearing at 500 Hz. COR findings demonstrating hearing amplification with hearing aids, indicating insufficient amplification to obtain good language development.

**Figure 15:**
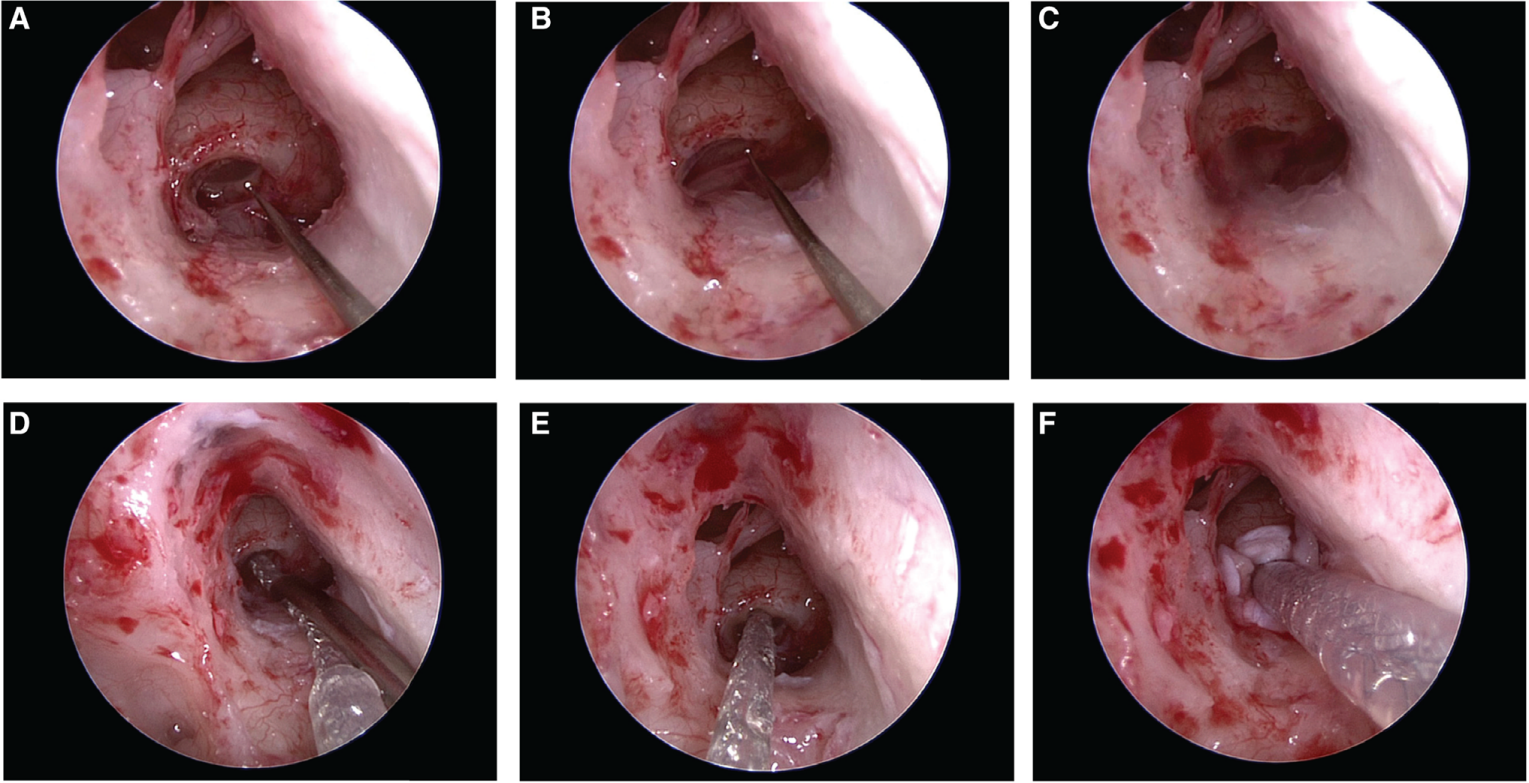
**A**: Endoscopic view of the electrode insertion procedure during cochlear implantation. The round window membrane was opened with a small pick. **B, C**: A cerebrospinal fluid (CSF) leakage was observed when opening the round window. **D, E**: The CSF leakage was easily stopped by the insertion of the electrode and **F**: shielding with fascia.

**Figure 16:**
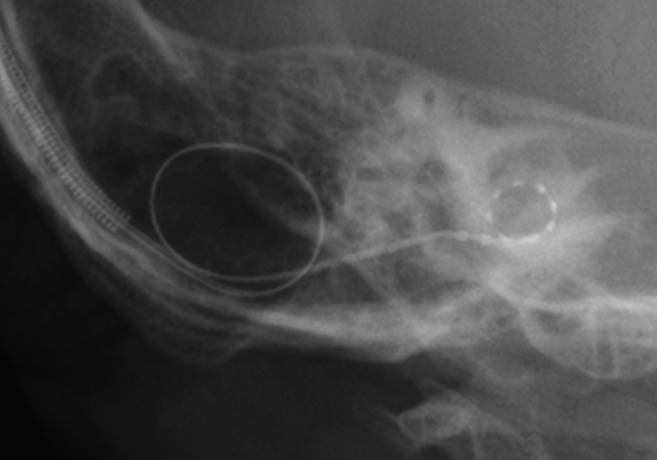
Postoperative X‐ray findings showing the full insertion of the electrode.

The patient (a 26‐month‐old girl) had compound heterozygous *SLC26A4* mutations (p.[K369E];[H723R]), and the parents were found to be carriers for the mutation (Fig. 14A). Her newborn screening tests, using automated ABR (AABR), found her hearing loss. ABR evaluated at the age of six months showed severe hearing loss (right: 70 dB, left: 80 dB). ASSR evaluated at 13 months showed severe hearing loss comparable with the ABR results and residual hearing at 500 Hz (Fig. 14D). CT scans showed EVA accompanied by cochlea hypoplasia (Mondini deformity) (Fig. 14B,C). COR evaluated at 18 months showed insufficient amplification to obtain good language development (Fig. 14E) and she received CI (MED‐EL CONCERTO) at two years of age. Due to cochlear hypoplasia, cerebrospinal fluid oozing was observed during CI surgery (Fig. 15), and a medium length electrode (FLEX24 electrode) was used to cover all frequencies in this case (Fig. 16). CI outcomes for this patient were favorable and she currently goes to a regular school.

In this case, genetic testing identified compound heterozygous mutations in the *SLC26A4* gene. Thereby, a clinician knew that she may experience profound hearing loss, which could potentially worsen. Amplification using hearing aids was found to be insufficient for good language development. Taken together with her etiology of hearing loss (intra‐cochlear etiology), these findings indicated that CI was the recommended intervention. Therefore, a wait‐and‐see strategy was identified as not a good option for this patient. The clinician was also able to provide information regarding the future possible appearance of other symptoms (vertigo and goiter) in association with the mutations in this gene.

### Stereocilia‐Related Genes

#### *CDH23*

**Figure 17:**
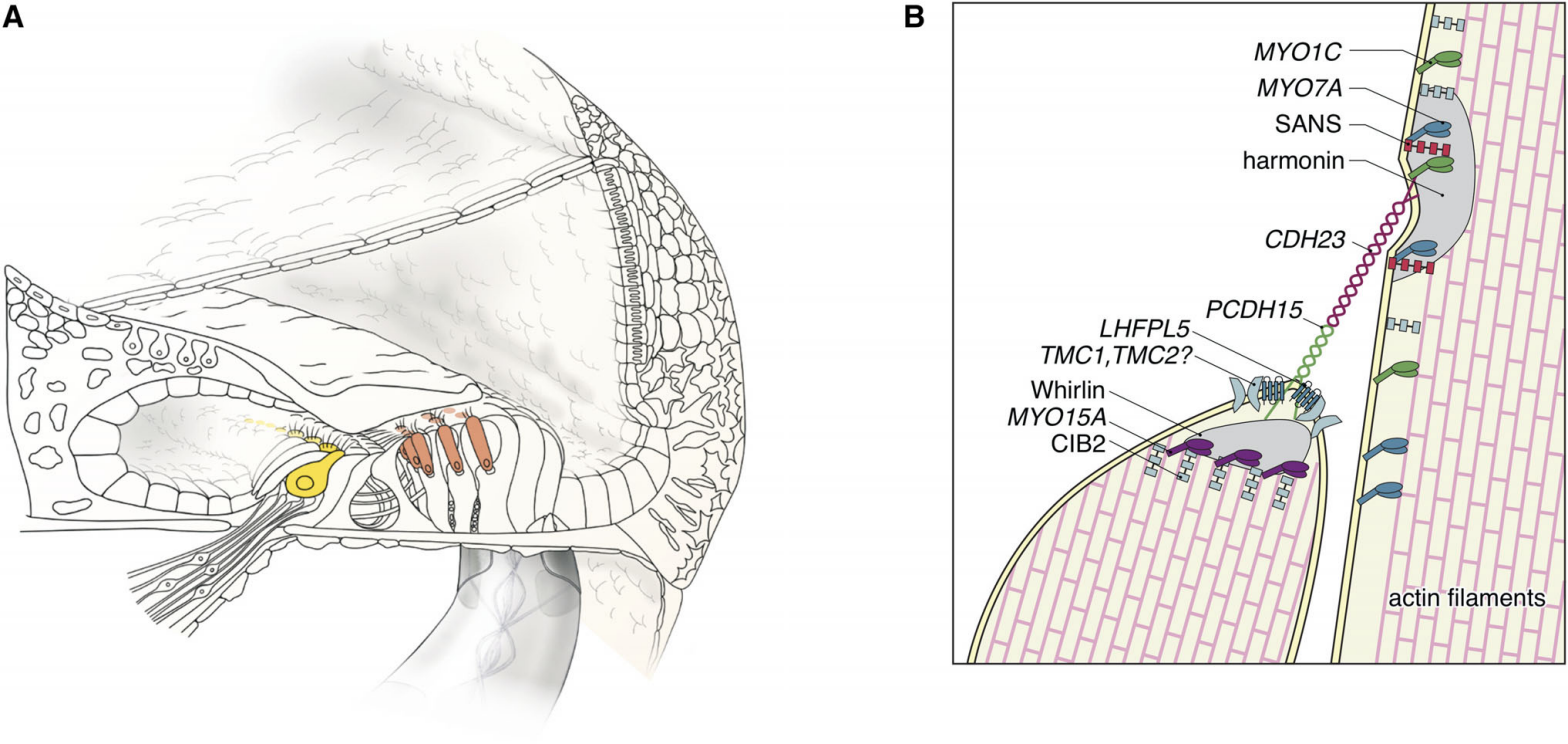
The cadherin 23 protein is an important component of the tip link that maintains the arrangement of stereocilia. **A**: The *CDH23* gene is expressed in inner and outer hair cells, predominantly. **B**: Schematic image of the tip‐link region of hair cell stereocilia (Nishio et al., 2015).

The gene *CDH23*, a member of the cadherin superfamily, encodes calcium‐dependent cell–cell adhesion glycoproteins, and is expressed in the inner and outer hair cells in the cochlea (Fig. 17). The encoded protein cadherin 23 is thought to interact with protocadherin 15 to form tip‐link filaments (Siemens et al., 2004; Söllner et al., 2004; Kazmierczak et al., 2007) (Fig. 17).

*CDH23* gene mutations may cause either Usher syndrome type 1D (USH1D) or non‐syndromic hearing loss (DFNB12). The phenotype range of DFNB12 is variable from congenital profound hearing loss to adult‐onset high‐frequency involved hearing loss. As shown in our past studies, *CDH23* mutations are often identified in patients having recessive inheritance, and one phenotypic feature is the presence of residual hearing (Miyagawa et al., 2012).

**Figure 18:**
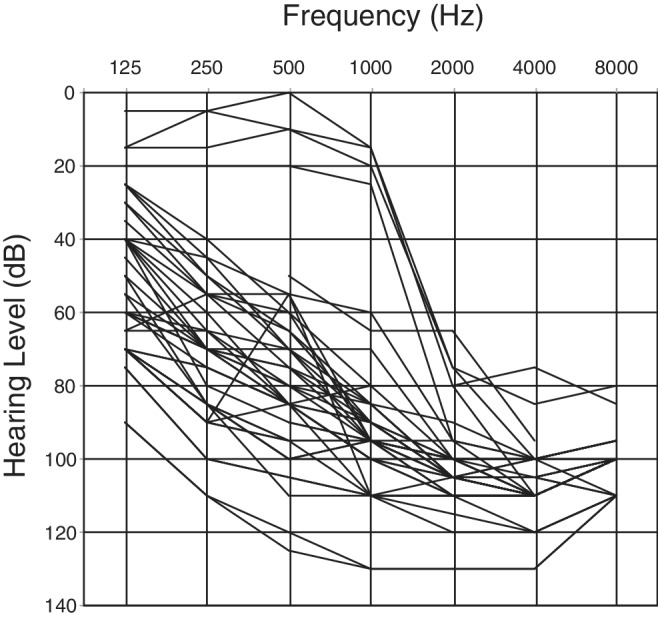
Overlapping audiograms of the patients with biallelic *CDH23* mutations, showing high‐frequency involved sensorineural hearing loss with residual hearing at the lower frequencies (Miyagawa et al., 2012).

**Figure 19:**
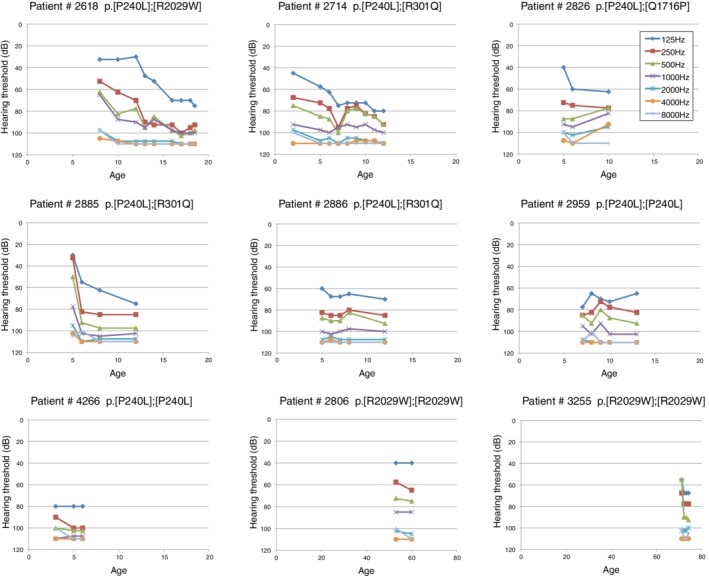
Hearing progression of patients with *CDH23* mutations. The high‐frequency portion had already worsened even in younger patients, and the low‐frequency portion deteriorated at later ages (Miyagawa et al., 2012).

It is well known that there are some genotype–phenotype correlations; i.e., patients with p.[P240L];[P240L] have greater hearing loss than do patients with the other mutations, tending to be congenital and severe. On the other hand, p.[R2029W];[R2029W] patients have a milder phenotype with middle age onset (Miyagawa et al., 2012). Overlapping audiograms of these patients typically show high‐frequency involved sensorineural hearing loss while retaining hearing at the lower frequencies (Fig. 18) (Miyagawa et al., 2012). Serial audiograms from the same patients show the progressive nature of the hearing loss caused by *CDH23* mutations (Fig. 19).

Regular CI has been applied for patients with insufficient amplification by hearing aids (Miyagawa et al., 2012) and, for the patients with residual hearing, EAS devices are a good therapeutic option (Usami et al., 2012; Moteki et al., 2017, 2018). A significant portion of EAS patients possess *CDH23* mutations (Moteki et al., 2017, 2018; Yoshimura et al., submitted). Therefore, it is extremely important to perform atraumatic CI surgery to preserve residual hearing for this particular category of patients. We have shown that hearing preservation can be achieved even with an electrode traversing the region of residual hearing (Usami et al., 2011).

For very young children, however, it is quite difficult to evaluate residual hearing. Residual hearing in the low frequencies cannot be measured by ABR. ASSR is currently available for the measurement of hearing levels at 500 or 250 Hz; however, these low‐frequency measurements are not reliable or convincing. As an adjunct to these hearing tests, genetic testing can help predict residual hearing at low frequencies. We have demonstrated that EAS can be successfully performed in very young patients with *CDH23* mutations, with the patients showing remarkable auditory performance after receiving EAS (Usami et al., 2012).

### Case: Estimation of Residual Hearing and Possible Associated Symptoms with Good Candidacy for EAS

**Figure 20:**
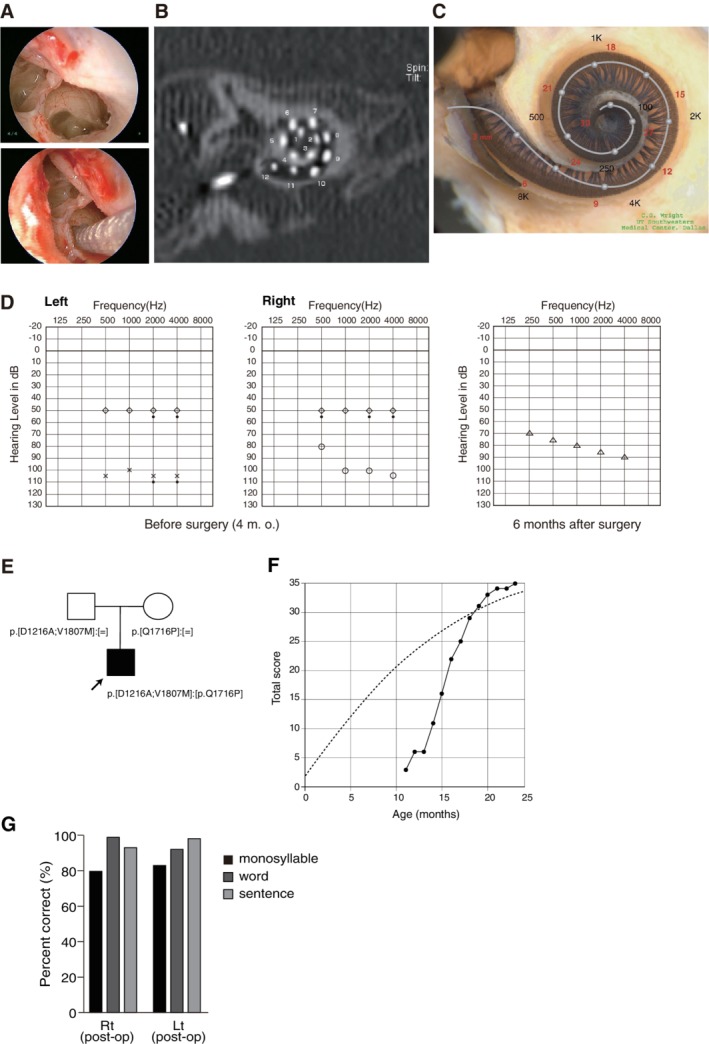
Findings for an EAS patient with *CDH23* mutations. **A**: Endoscopic view of a round window insertion, **B**: postoperative CT findings, **C**: imaging with the putative location of the electrode and the referential tonotopic map, **D**: preoperative ASSR findings and postoperative COR audiogram findings. **E**: Pedigree and mutations. **F**: Auditory behavioral development assessed by LittlEARS auditory questionnaire, indicating rapid improvement in auditory behavior reaching the curve of normally developed children (A–F; Usami et al., 2012). **G**: Speech perception score at the age of seven showing good outcome.

This case was a one‐year‐old boy with a *CDH23* mutation. This patient had compound heterozygous mutations (p.[D1216A;V1807M];[Q1716P]), and the parents were determined to have been the mutation carriers (Fig. 20E). Newborn hearing screening found his hearing loss through the use of automated ABR. No response was obtained by ABR. ASSR evaluated at the age of three months showed some residual hearing at 500 Hz in the right ear (Fig. 20D). His hearing level wearing hearing aids was not adequately amplified for normal language development. Based on the above together with his intra‐cochlear etiology, he underwent CI for his left ear (MEDEL PULSAR CI100/ standard electrode) at nine months of age. The parents also requested right ear CI. Based on ASSR evaluation, residual hearing was noted in the right ear. For very young children, it is very difficult to estimate residual hearing. In addition to the ASSR evaluation, genetic testing gives us very important information. In this child, we found *CDH23* mutations. Our series of studies indicated that patients with *CDH23* mutations have some residual hearing (Wagatsuma et al., 2007; Miyagawa et al., 2012). The combination of ASSR and genetic testing is a powerful tool to estimate audiogram configuration for very young children. Due to possible low‐frequency residual hearing, we used a more atraumatic electrode (MEDEL PULSAR CI100/FLEXsoft electrode) (Fig. 20A–C)). Residual hearing measured by COR was well preserved one year after CI (Fig. 20D). Speech perception test at Age 10 was dramatically improved (monosyllable: rt 80%, lt 83%; word: rt 100%, lt 92%; sentence: rt 94%, lt 98%) and the child currently goes to a regular school (Fig. 20G).

**Figure 21:**
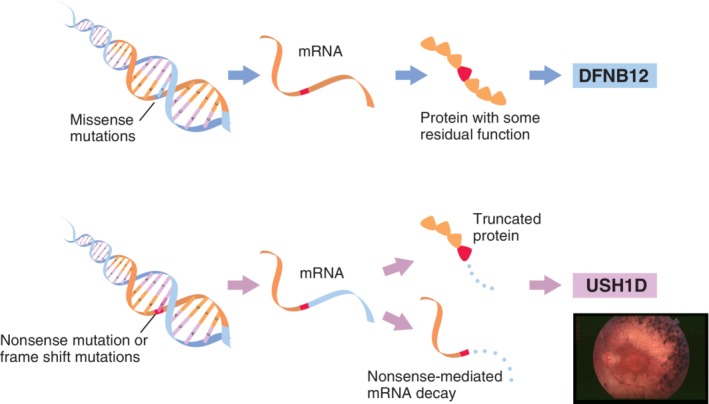
Phenotype–genotype correlation in the *CDH23* gene. In general, missense mutations of the *CDH23* gene are associated with non‐syndromic hearing loss, whereas nonsense or frame shift mutations cause Usher syndrome.

The spectrum of diseases caused by *CDH23* mutations include non‐syndromic hearing loss (DFNB12) and USH1D (Bork et al., 2001; Astuto et al., 2002; Miyagawa et al., 2012; Yoshimura et al., 2014). Would it be possible to obtain any information from genetic testing? The patient was not indicative for Usher syndrome. He did not have vision deterioration or problems with vestibular function, but at that time was also too young for ophthalmologic examinations. However, some patients who are diagnosed with non‐syndromic hearing loss may sometimes have associated symptoms later (Yoshimura et al., 2013). Genetic testing can estimate such associated symptoms at a very early stage. A series of studies indicated that missense mutations (found in this case) may cause non‐syndromic hearing loss, whereas nonsense or frame shift mutations which produce truncated proteins may cause the Usher phenotype (Fig. 21). Thus, the risk of retinitis pigmentosa in this case may be not so high, but the parents must pay some attention to his visual symptoms.

### Case: Adult‐Onset Progressive High‐Frequency Involved Hearing Loss with Good Candidacy for EAS

**Figure 22:**
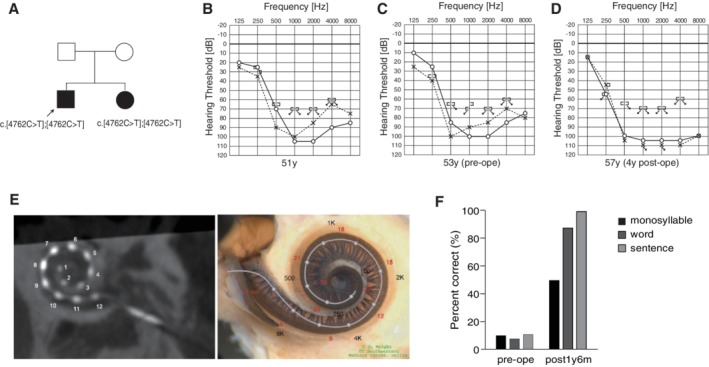
Findings for an EAS patient with homozygous *CDH23* mutations. **A**: Pedigree of the patient with *CDH23* mutations. **B**: Preoperative audiogram at age 51. **C**: Preoperative audiogram at age 53. His hearing showed deterioration at 500 Hz. **D**: Hearing thresholds four years after cochlear implantation. Residual hearing in lower frequencies was well preserved. **E**: Postoperative CT imaging and estimated electrode position. **F:** Speech perception score at age 54 showing good outcome.

The patient noticed hearing loss in his late 30s, which thereafter gradually progressed. He felt some inconvenience around age 45, and started wearing hearing aids. He visited our hospital at age 51, and was found to have homozygous *CDH23* mutations (p.[R1588W];[R1588W]) (Fig. 22A). He had very good residual hearing at 125 and 250 Hz. Considering the progressive nature of hearing loss (Fig. 22B,C), we chose a longer electrode in order to cover the low frequency region, and he received CI (MEDEL Synchrony/FLEX28 electrode) at the age of 53 for the left ear. His low‐frequency hearing after the implantation was completely preserved (Fig. 22D). Therefore, he uses the natural residual hearing combined with ES (electric stimulation) only, and does not require any acoustic amplification at this moment. Postoperative CT scans indicated that although the tip of the electrode reached the 250 Hz region, his low tone residual hearing was well preserved (Fig. 22E). As shown in the previous literature (Usami et al., 2011), hearing preservation can be achieved in the presence of an electrode beneath the basal membrane covering the residual hearing region. Speech perception test results were dramatically improved (monosyllable: 50%; word: 88%; sentence: 100%) (Fig. 22F).

#### *MYO7A and MYO15A*

**Figure 23:**
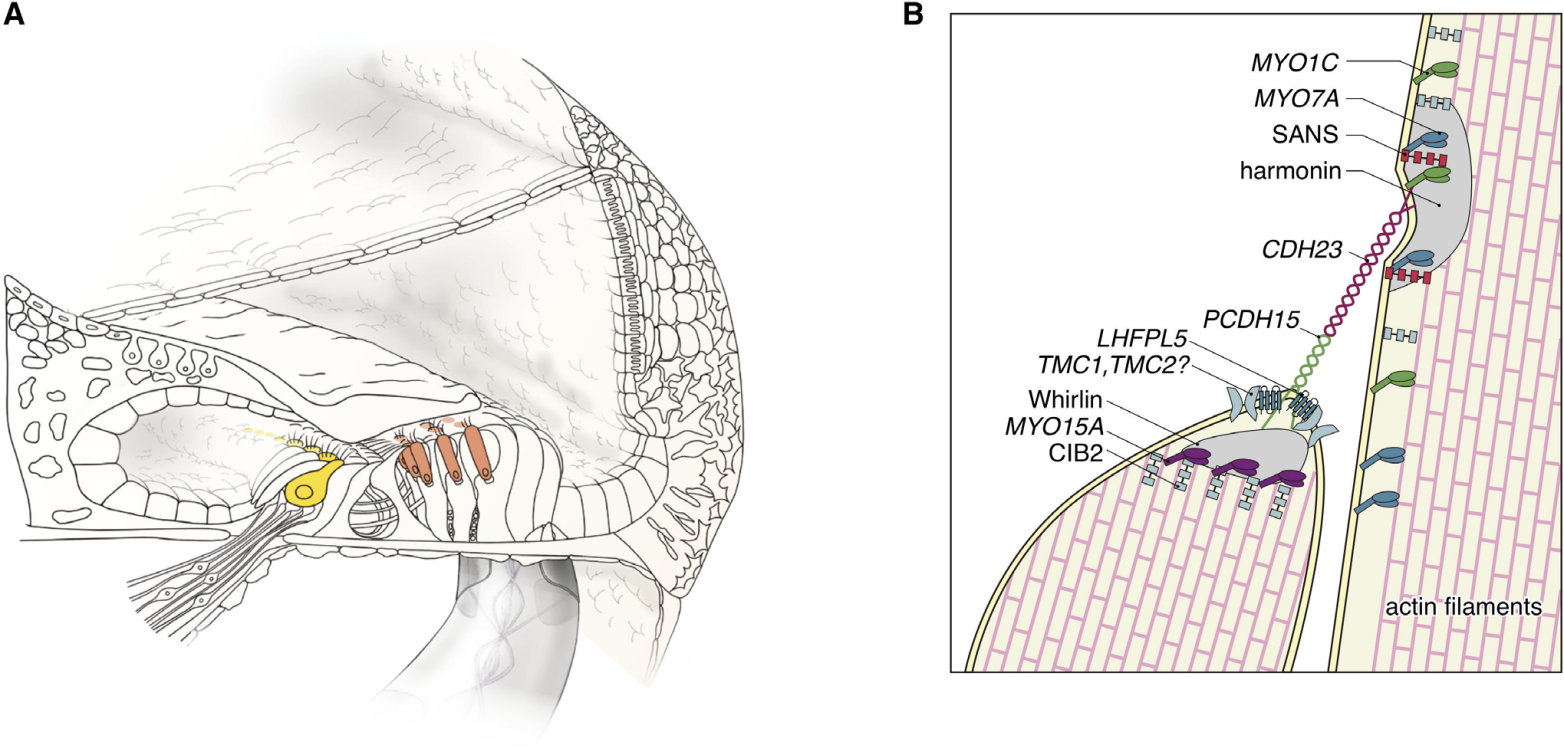
The myosin VIIA and XVA proteins are important components of stereocilia. **A**: *MYO7A* and *MYO15A* are predominantly expressed in inner and outer hair cells. **B**: Schematic image of the tip‐link region of hair cell stereocilia (Nishio et al., 2015).

*MYO7A* and *MYO15A* encode myosin VIIA and XVA, members of the unconventional myosin superfamily of proteins (Krendel and Mooseker, 2005). Myosin VIIA is localized in stereocilia and plays the role of an anchor (Hasson et al., 1997; Wolfrum et al., 1998; Boeda et al., 2002). Myosin XVA plays an indispensable role in the graded elongation of stereocilia and actin organization in hair cells of the cochlea, and therefore the function of these myosin in the hair cells in the cochlea is crucial for transducing sound information into the primary afferent neuron (Belyantseva et al., 2003) (Fig. 23). Myosin genes are known to be responsible for 10 types of syndromic as well as non‐syndromic hearing loss (*MYO7A*, DFNA11/DFNB2/USH1B; *MYH9*, DFNA17; *MYH14*, DFNA4; *MYO6*, DFNA22/DFNB37, *MYO3A*, DFNB30; *MYO15A*, DFNB3). *MYO7A* mutations may cause various phenotypes ranging from non‐syndromic hearing loss (DFNA11/DFNB2) to Usher syndrome (USH1B) (Weil et al., 1995; Liu et al., 1997). *MYO15A* mutations are known to be responsible for DFNB3 (Wang et al., 1998) and further phenotypic study indicated that there are two types of hearing impairment phenotypes: (1) prelingual onset and severe or profound hearing loss or (2) a milder phenotype with postlingual onset and progressive hearing loss (Miyagawa et al. 2015a).

There have been several studies describing outcomes of CI/EAS for patients with *MYO7A* and *MYO15A* mutations. As in the three cases being introduced in this review, all previous reports indicated satisfactory outcomes after CI/EAS (Miyagawa et al., 2013, 2015a; Chang et al., 2015, 2018; Jung et al., 2017; Liu et al., 2019).

### Case: DFNA11 Caused by a *MYO7A* Mutation

#### *Late‐onset progressive high‐frequency involved hearing loss with good candidacy for EAS*

**Figure 24:**
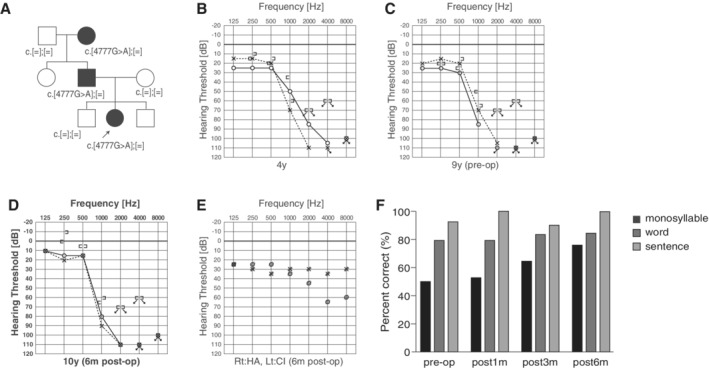
Findings for an EAS patient with *MYO7A* mutations. **A**: Pedigree and mutations. **B**: Hearing thresholds at four years of age. **C**: Hearing thresholds at nine years of age (pre‐op). Hearing at 1,000 Hz and 2,000 Hz was deteriorated. **D**: Hearing thresholds six months after cochlear implantation. Residual hearing in lower frequencies was well preserved. **E**: Hearing thresholds with CI and hearing aid. **F**: Speech perception test results were dramatically improved after CI.

A 10‐year‐old girl. She passed her newborn hearing screening. Hearing loss was suspected at age 4, and she visited our hospital. At the age of 9, hearing deterioration was observed at 1 kHz. She was not satisfied with hearing aids, and received EAS (MED‐EL Synchrony/FLEX24) for the left ear at the age of 10. Residual hearing was completely preserved six months after the surgery. She uses the natural residual hearing combined with ES, and does not require any acoustic amplification at low frequency. Speech perception test results were dramatically improved (monosyllable: 77%; word: 84%, sentence: 100%) (Fig. 24).

### Case: DFNB3 Caused by *MYO15A* Mutations

#### *Congenital progressive hearing loss with good candidacy for CI*

**Figure 25:**
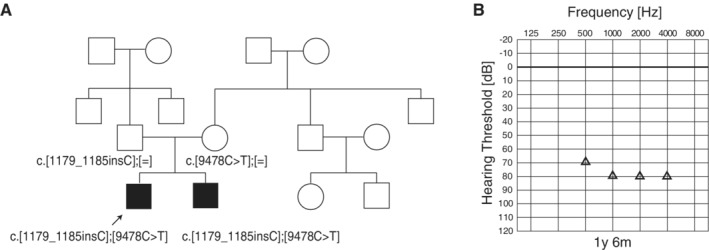
The CI patients with *MYO15A* mutations. **A**: The pedigree and results of genetic testing. **B**: COR audiogram findings at one year and six months of age (Miyagawa et al., 2013).

This case is a five‐year‐old boy. Newborn hearing screening indicated hearing loss. ASSR and COR showed progressive hearing loss. The patient had compound heterozygous *MYO15A* mutations (c.[9478C>T];[1179_1185insC]) inherited from the parents (Fig. 25A). Both mutations were predicted to be pathologic. Although hearing aids helped with some language development, progressive hearing loss remained. As amplification alone was determined to be inadequate, CI surgery was performed for the left ear (MEDEL PULSAR CI100/standard electrode) when he was four years nine months. Three months after CI use of language was developed (Scores of IT‐MAIS: 16/40.25/40, LittlEARS:28.33). However, in order to assess the final outcome, long‐term follow up is required.

### Case: DFNB3 Caused by *MYO15A* Mutations

#### *Congenital progressive high‐frequency involved hearing loss with good candidacy for EAS*

**Figure 26:**
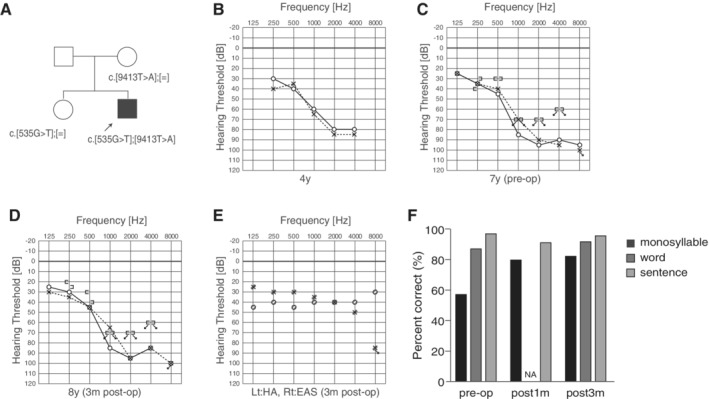
Findings for an EAS patient with *MYO15A* mutations. **A**: Pedigree and mutations. **B**: Hearing thresholds at four years at age assessed by COR testing. **C**: Hearing thresholds at seven years at age (pre‐op). **D**: Hearing thresholds three months after cochlear implantation. Residual hearing in lower frequencies was well preserved. **E**: Hearing thresholds with EAS and hearing aid. **F**: Speech perception test results were dramatically improved after EAS.

An eight‐year‐old boy. Bilateral hearing loss was detected through newborn hearing screening. He visited our hospital at three months old. Due to high‐frequency hearing loss, he started wearing hearing aids at eight months old. He felt some inconvenience and received right EAS at the age of eight. Speech perception test results were dramatically improved three months after EAS (monosyllable: 82%; word: 92%, sentence: 96%) (Fig. 26).

### *ACTG1*

**Figure 27:**
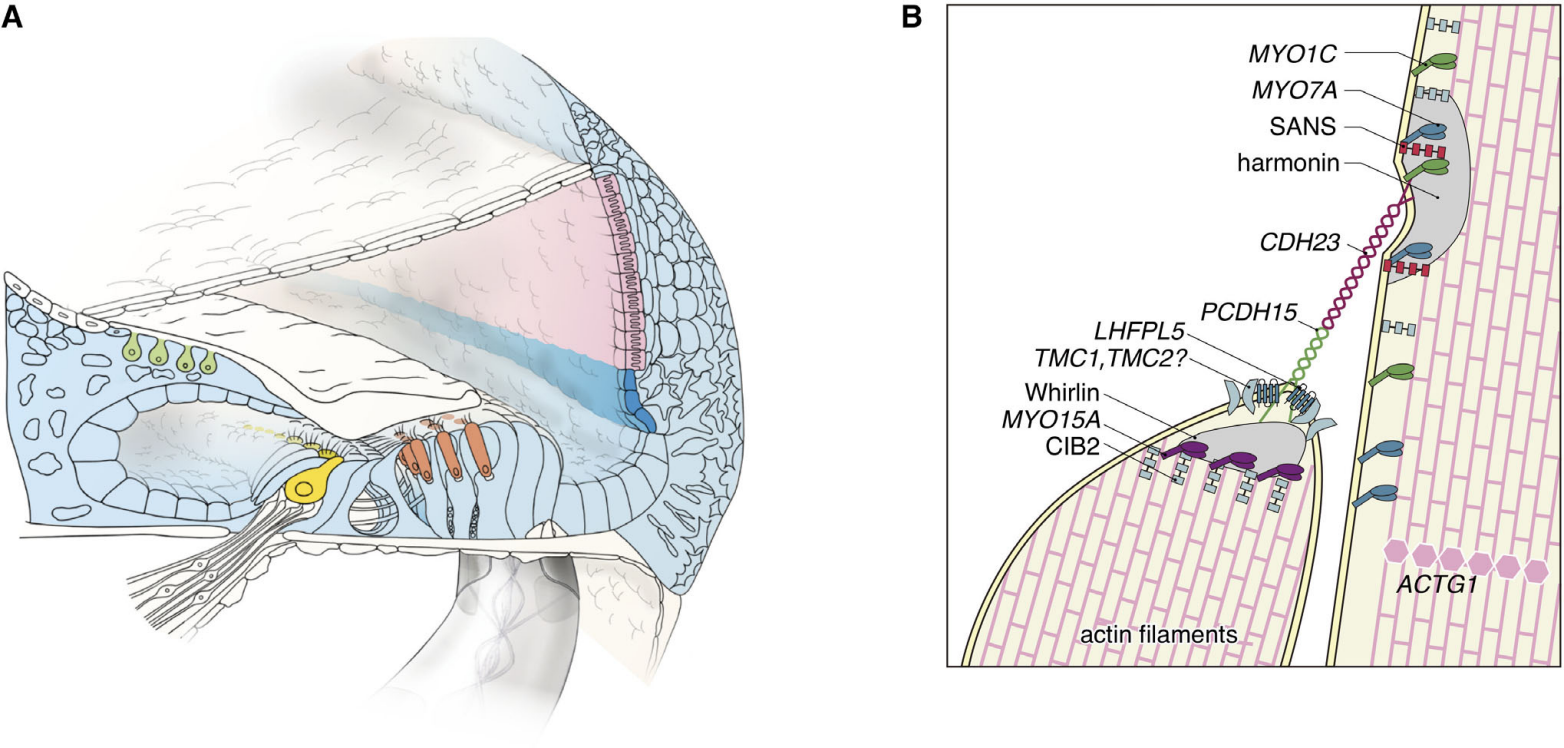
**A**: Immunocytochemical localization of gamma‐actin in the cochlea. **B**: Gamma‐actin is expressed in most cochlear cells. In hair cell stereocilia, gamma‐actin is localized in the gap regions of F‐actin filaments and may act as an F‐actin gap connection (modified from Nishio et al., 2015).

*ACTG1* encodes γ‐actin, the predominant actin isoform in the inner and outer hair cells in the cochlea. Within the hair cells, γ‐actin is an important component of the cuticular plate, adherens junctions, and stereocilia (Khaitlina, 2001; Belyantseva et al., 2009) (Fig. 27). Since the expression of *ACTG1* is located within the cochlea, comparatively good outcomes for CI/EAS can be expected. In fact, our studies demonstrated that EAS was an effective therapeutic intervention for patients with *ACTG1* mutations (Miyagawa et al., 2013, 2015b).

### Case: DFNA20/26 Caused by *ACTG1* Mutations

#### *Late‐onset progressive hearing loss with good candidacy for EAS*

**Figure 28:**
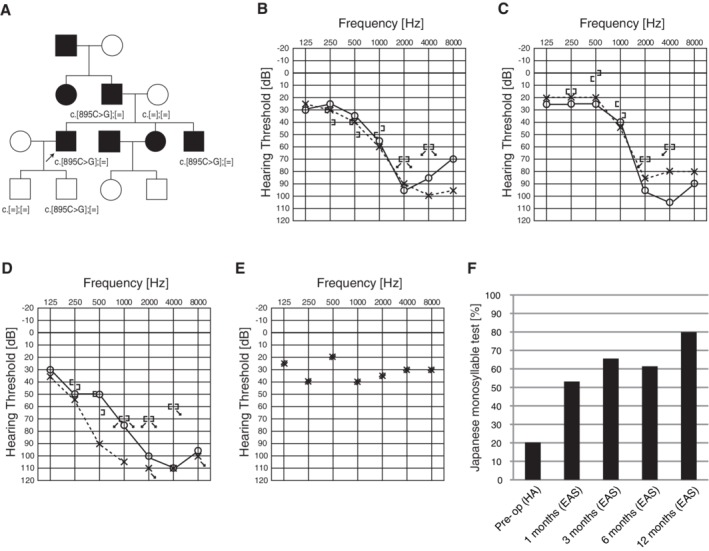
The EAS patient with a *ACTG1* mutation. **A**: Pedigree and mutations. Pedigree is compatible with autosomal dominant hearing loss. **B**: Preoperative audiogram. **C**: Audiogram of brother. **D**: Postoperative audiogram (six months after EAS). **E**: Hearing thresholds with EAS. **F**: Japanese monosyllable test results showing dramatic improvement with EAS (Miyagawa et al., 2015b).

A 39‐year‐old male. His high‐frequency involved hearing loss was first diagnosed through a primary school physical examination at the age of 12 (Miyagawa et al., 2013). He noticed progression of hearing loss and episodes of tinnitus at around age 20. He started wearing a hearing aid at age 33 and visited our hospital at age 34. He had a heterozygous *ACTG1* mutation, c.895C>G (p.L299V), and his father, brother, and younger son carried the same mutation. Due to progressive hearing loss and insufficient amplification by hearing aids, he received EAS at age 39. His brother showed a similar audiogram configuration from around 15 years old. Residual hearing was preserved and monosyllable tests showed dramatic improvement. Speech perception test results were dramatically improved from 20% to 80% one year after receiving the EAS (Fig. 28).

#### *TMPRSS3*

*TMPRSS3* is known to be a member of the Type II Transmembrane Serine Protease family. Important for cochlear neurons maintenance and development, the processing of proneurotrophins is thought to be regulated by *TMPRSS3* (Guipponi et al., 2002). Previous studies report *TMPRSS3* to cause two different hearing loss phenotypes, (1) DFNB10: congenital or early childhood onset, with severe and prelingual hearing impairment, and (2) DFNB8: a later‐onset ski‐slope type audiogram and progressive postlingual hearing impairment.

In mice with mutations in *Tmprss3*, hair cells in the organ of Corti were found to be deteriorated at the start of the basal turn, and progressing toward the apex (Fasquelle et al., 2011). This progressive degeneration pattern observed in the mouse model is consistent with the human phenotype, which has high‐frequency involved progressive hearing loss.

Based on the reported phenotype, *TMPRSS3* should be considered as a frequent etiology for potential EAS patients. However, *TMPRSS3* CI patient outcomes remain disputed (Elbracht et al., 2007; Weegerink et al., 2011; Eppsteiner et al., 2012; Miyagawa et al., 2013, 2015c). A majority of the reported CI cases (13 out of 15 from the literature) had favorable outcomes, but two cases reported by Eppsteiner et al. (2012) showed poorer performances.

**Figure 29:**
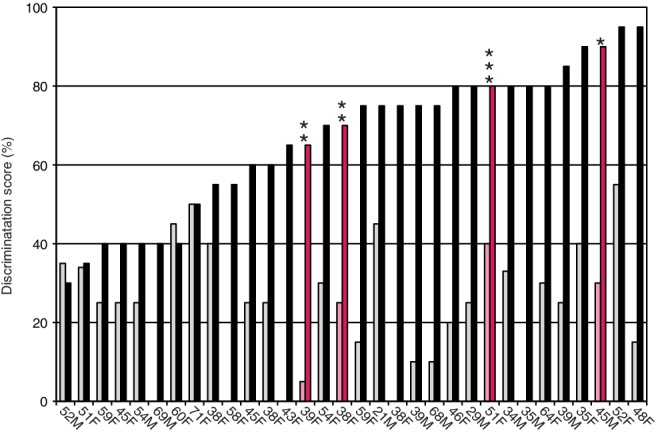
Speech discrimination scores (using the 67S Japanese monosyllable test, 70 dBSPL) preoperatively (gray) and at 12 months after the initial EAS (black). The four patients with *TMPRSS3* mutations (pink) showed significant improvement (Miyagawa et al., 2015c).

**Figure 30:**
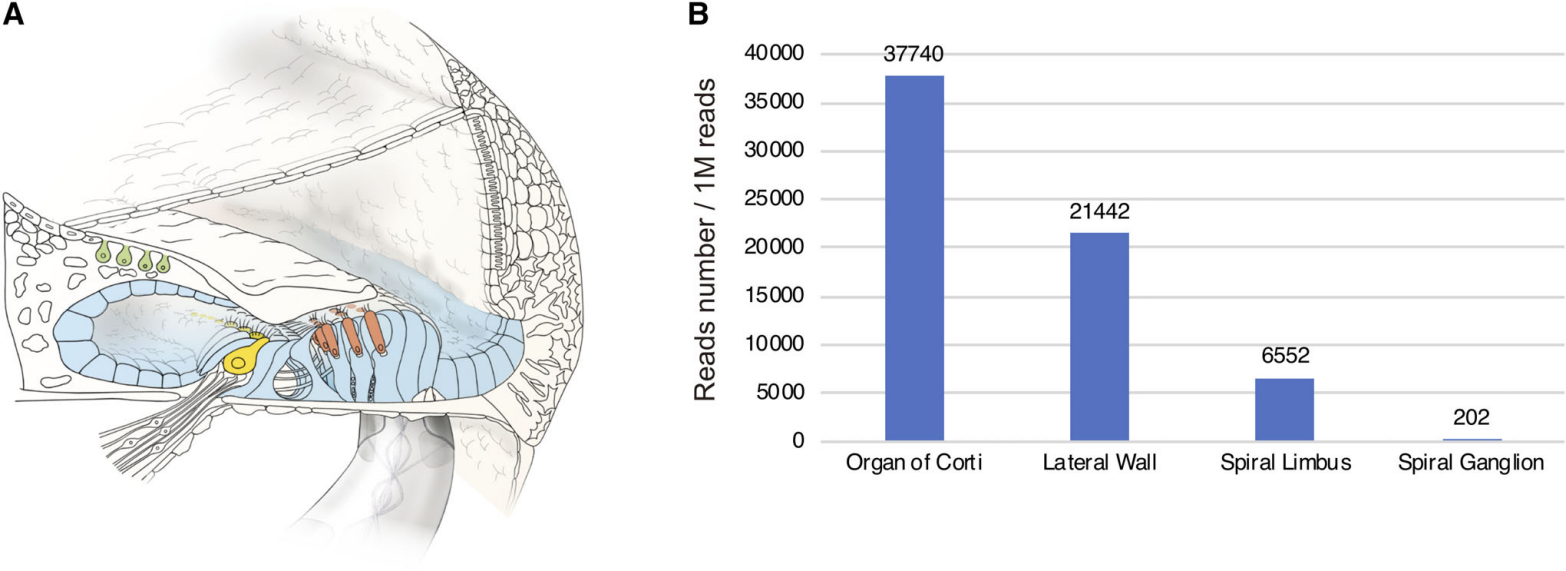
**A**: *TMPRSS3* is predominantly expressed in inner and outer hair cells, supporting cells, inner and outer sulcus cells, and interdental cells. **B**: *Tmprss3* gene expression in the mouse cochlea and spiral ganglion (modified from Nishio et al., 2017).

*TMPRSS3* expression is also found in the spiral ganglion, and previous literature reported ganglion cell death in mutants (Fasquelle et al., 2011; Eppsteiner et al., 2012). Therefore, it is possible that poor performance is related to afferent neuronal cell loss. However, the majority of cases, including three EAS cases evaluated by our group, had satisfactory outcomes, promoting the idea that CI and/or EAS has good potential as a therapeutic option (Fig. 29). Our recent gene expression study, in which the *Tmprss3* gene was shown to be predominantly expressed within the cochlea (Nishio et al., 2017), supported our clinical data (Fig. 30).

CI and/or EAS may be elected for therapy. In particular, a patient with high‐frequency hearing loss is a good candidate for EAS, as with the previously described patient. Clinicians should recognize that patients with mutations in *TMPRSS3* may have progressive hearing loss. Proper intervention should be offered during follow‐up periods for these patients.

### Case: DFNB8 Caused by *TMPRSS3* Mutations

#### *Late‐onset progressive hearing loss with good candidacy for EAS*

**Figure 31:**
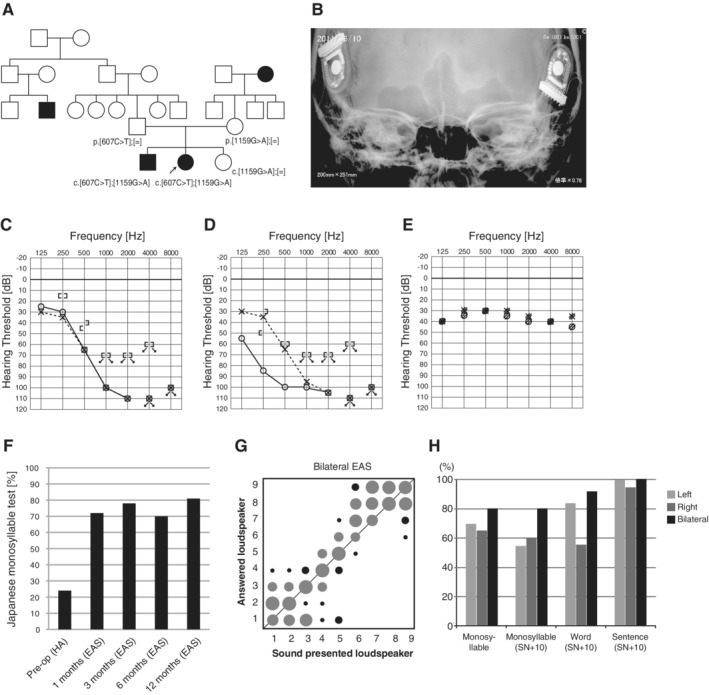
The EAS patient with *TMPRSS3* mutations. **A**: The patient has compound heterozygous *TMPRSS3* mutations, and the parents were found to be carriers for these mutations. **B**: X‐ray imaging after bilateral EAS. **C**: Preoperative audiogram. **D**: Postoperative audiogram (left: 24 months after first EAS, right: four months after second EAS). **E**: Hearing threshold with bilateral EAS. **F**: Japanese monosyllable test (65 dB SPL in quiet) showing dramatic improvement with bilateral EAS (A‐F; Miyagawa et al., 2013). **G**: After bilateral EAS, the sound localization ability with bilateral EAS improved. **H**: Speech perception in quiet and noise improved (G, H; Moteki et al., 2015).

A 40‐year‐old female (Usami et al., 2011; Miyagawa et al., 2013, 2015c; Moteki et al. 2015). Hearing loss was first reported during grade school after a general student screening. As her hearing loss was progressive, she started to wear hearing aids at the age of 25. However, due to insufficient hearing, EAS (MED‐EL PULSAR/FLEX24) was applied at the ages of 38 and 39 (Fig. 31B). The patient had compound heterozygous *TMPRSS3* mutations c.[607C>T];[1159G>A] (p.[Q203X];[A387T]) (Fig. 31A). Preservation of residual hearing allowed for acoustic amplification and, with bilateral EAS, her hearing was measured as 30dBHL (Fig. 31C–E). An impressive gain was found on the Japanese monosyllable test (65 dB SPL in quiet), where after only one year after EAS she improved from 24% to 81% (Fig. 31F). After bilateral EAS, the sound localization ability with bilateral EAS improved, and speech perception in quiet and noisy environments improved (Fig. 31G,H).

#### *The m.1555A>G mitochondrial mutations*

The m.1555A>G mutation in the mitochondrial 12S ribosomal RNA gene is the most common mitochondrial mutation associated with hearing loss (Usami et al., 2000a, b; Yano et al., 2014). This form of hearing loss is usually associated with aminoglycoside exposure, but some cases without a history of aminoglycoside exposure have been observed (Usami et al., 2000b). Patients with no history of aminoglycoside exposure usually experience a milder degree of hearing loss compared to those with aminoglycoside exposure (Usami et al., 2000b). This mutation was first identified after studies of familial aggregation of aminoglycoside‐induced hearing loss indicated an exclusively maternally transmitted constitutional susceptibility to cochlear damage caused by aminoglycoside antibiotics exists in some families. Genetic analysis revealed that this hereditary susceptibility is due to a m.1555A>G point mutation in the mitochondrial 12S ribosomal RNA gene (Prezant et al., 1993). The m.1555A>G point mutation, which leads to the structure being more similar to the bacterial ribosomal RNA structure, increases the binding properties of aminoglycoside (Böttger., 2010).

Individuals with the m.1555A>G mitochondrial mutation are susceptible to hearing loss from the use of aminoglycoside antibiotics. This mutation is commonly involved in hearing loss among the Japanese population, affecting 3% of sensorineural hearing loss patients (Usami et al., 2000a). Excellent auditory performance after CI has been reported in patients with this mutation and should be considered as therapy for this population (Tono et al., 1998). Recently, we have reported the application of EAS to the treatment of a patient with high‐frequency involved hearing loss (Usami et al., 2012).

### Case: Non‐syndromic Hearing Loss Associated with m.1555A>G Mitochondrial Mutations

#### *Late‐onset progressive hearing loss with good candidacy for EAS*

**Figure 32:**
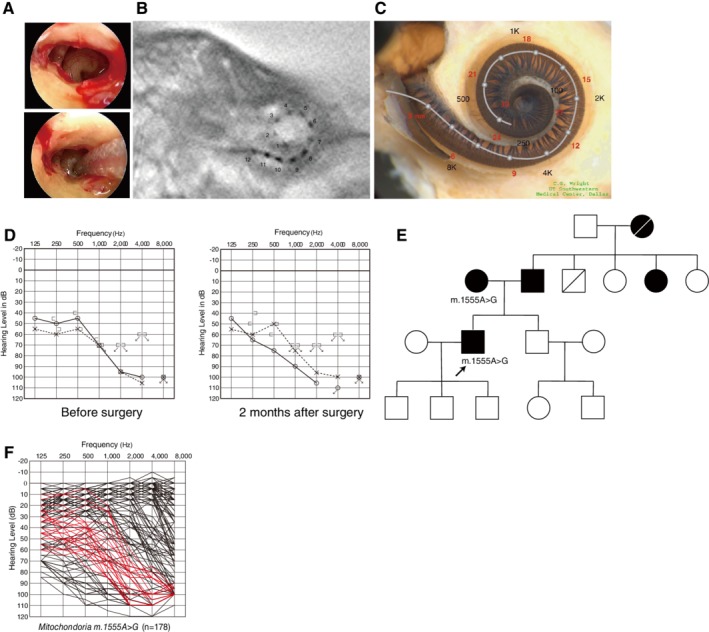
Findings for an EAS patient with the m.1555A>G mitochondrial mutation. **A**: Endoscopic view of round window insertion. **B**: Postoperative X‐ray findings. **C**: Imaging with putative location of electrode and the referential tonotoic map. **D**: Preoperative and postoperative audiograms. **E**: Pedigree and the subjects with the mitochondrial m.1555A>G mutation (Usami et al., 2012). **F**: Overlapping audiograms of patients with the m.1555A>G mutation, showing high‐frequency involved sensorineural hearing loss with residual hearing at the lower frequencies (Usami et al., 2010).

A 52‐year‐old male with the m.1555A>G mitochondrial mutation (Usami et al., 2012). He did not have any history of aminoglycoside antibiotics exposure. After age 38, hearing loss was noticed. Hearing aids were adopted, but audiograms indicated progressive hearing loss. The electrode MEDEL PULSAR CI100/FLEX24 was implanted as to be less traumatic for lower frequency residual hearing (Fig. 32A–C). After two months, tests indicated well preserved residual hearing (Fig. 32D). Pedigree analysis indicated autosomal dominant hearing loss, with parental hearing loss as well (Fig. 32E). The m.1555A>G mitochondrial mutation was detected in the patient and his mother. After two months from CI, increased hearing to 30 dBHL was measured. His speech perception score was improved four years after EAS (Japanese speech perception test CI2004: monosyllable; 55%, word; 84%, sentence; 96%).

#### *OTOF*

**Figure 33:**
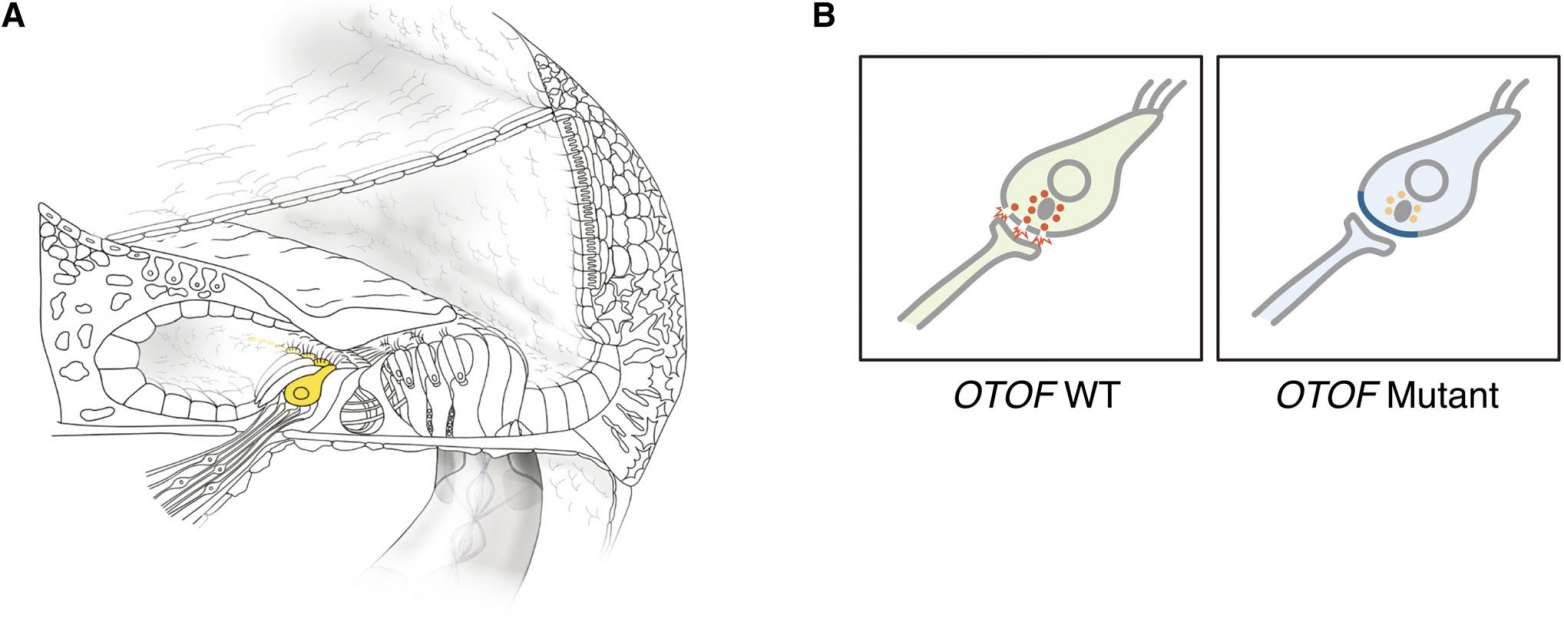
**A**: *OTOF* is predominantly expressed in inner hair cells. **B**: Otoferlin plays a crucial role in synaptic exocytosis at ribbon synapses. Mutations of the *OTOF* gene cause deficient or absent exocytosis from inner hair cells, but outer hair cell' function is preserved, and show an auditory neuropathy phenotype (synaptopathy).

Auditory neuropathy (AN) is hearing loss derived from the dysfunction of auditory signals downstream of and/or including the inner hair cells. It is marked by loss of ABR signal yet retention of OAE and/or cochlear microphonics (CM). Now known as “auditory neuropathy spectrum disorder (ANSD),” AN was renamed in 2008 due to being a spectrum of associated symptoms (Roush et al., 2011 for review). Although the exact percentage of non‐syndromic ANSD is unclear, responsible genes continue to be identified, with mutations in the *AUNA1*, *OTOF*, *PJVK*, and *GJB2* genes as well as in mitochondrial 12S rRNA reported to cause non‐syndromic ANSD (Manchaiah et al., 2011). The *OTOF* gene (DFNB9) is predominantly expressed in the cochlear inner hair cells, and is essential for synaptic exocytosis at their ribbon synapses (Roux et al., 2006). Therefore, mutations in the *OTOF* gene mutation result in dysfunction in the signal transmission between the inner hair cells and neurons (Fig. 33).

*OTOF* mutations are known to cause prelingual hearing loss, and severe‐to‐profound non‐syndromic hearing loss (Iwasa et al. 2013, 2019). OAEs have generally disappeared by two years of age; therefore, many patients are diagnosed with non‐syndromic sensorineural hearing loss (NSHL). Due to these studies, required screening for *OTOF* mutations is suggested for the patients diagnosed with ANSD and, furthermore, ARNSHL cases should be included as well. It should be borne in mind that NHS using OAE failed to detect ANSD, indicating that infants should undergo OAE testing as well as ABR. In a majority of cases, patients with *OTOF* mutations have been shown to have effective treatment by CI (Rodriguez‐Ballesteros et al., 2003; Rouillon et al., 2006; Wu et al., 2011; 2018; Zhang et al., 2013, 2016; Chen et al., 2018) due to good preservation of the spiral ganglions and auditory nerves. For this reason, a favorable result is anticipated for CI when an *OTOF* mutation is determined to be causative in a deaf patient, and this demonstrates the value of identifying genetic mutations in patients.

### Case: DFNB6 Caused by *OTOF* Mutations

#### *Congenital profound hearing loss with a positive OAE response*

**Figure 34:**
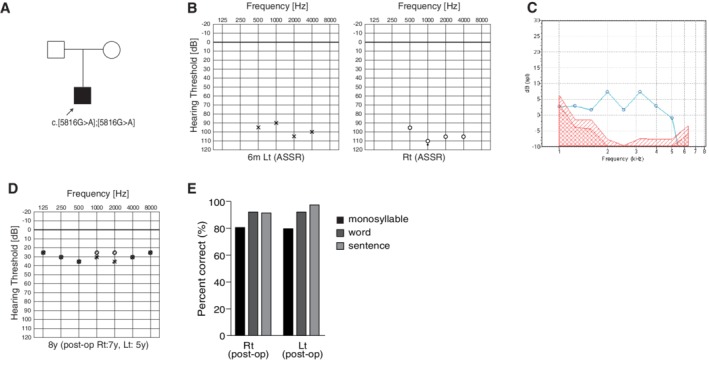
The CI patient with *OTOF* mutations. **A**: The patient has homozygous *OTOF* mutations. **B**: Preoperative ASSR findings. **C**: This patient showed a clear OAE response. **D**: Hearing threshold with bilateral CI. **E**: Japanese Speech perception score after CI showing a favorable outcome.

A seven‐year‐old boy with *OTOF* mutations. Newborn screening identified hearing loss using AABR. No response was obtained by ABR or ASSR (Fig. 34B,C), but the OAE response was normal. This patient had *OTOF* homozygous mutations (p.[R1172Q];[R1172Q]) (Fig. 34A). Based on the intra‐cochlear etiology, he received bilateral sequential cochlear implants (MEDEL CONCERTO/FLEXsoft electrode) at the age of one year three months for the right ear, and three years three months for the left ear. In this case, genetic testing gives us very important information as the etiology is located in the inner hair cells, not in the spiral ganglion. The combination of OAE and genetic testing is a powerful tool to estimate etiologies for very young ANSD children (Fig. 34B,C). Speech perception test at age 6 was dramatically improved (monosyllable: rt 80%, lt 80%; word: rt 92%, lt 92%; sentence: rt 91%, lt 97%) and he currently goes to a regular school (Fig. 34E).

#### *LOXHD1*

**Figure 35:**
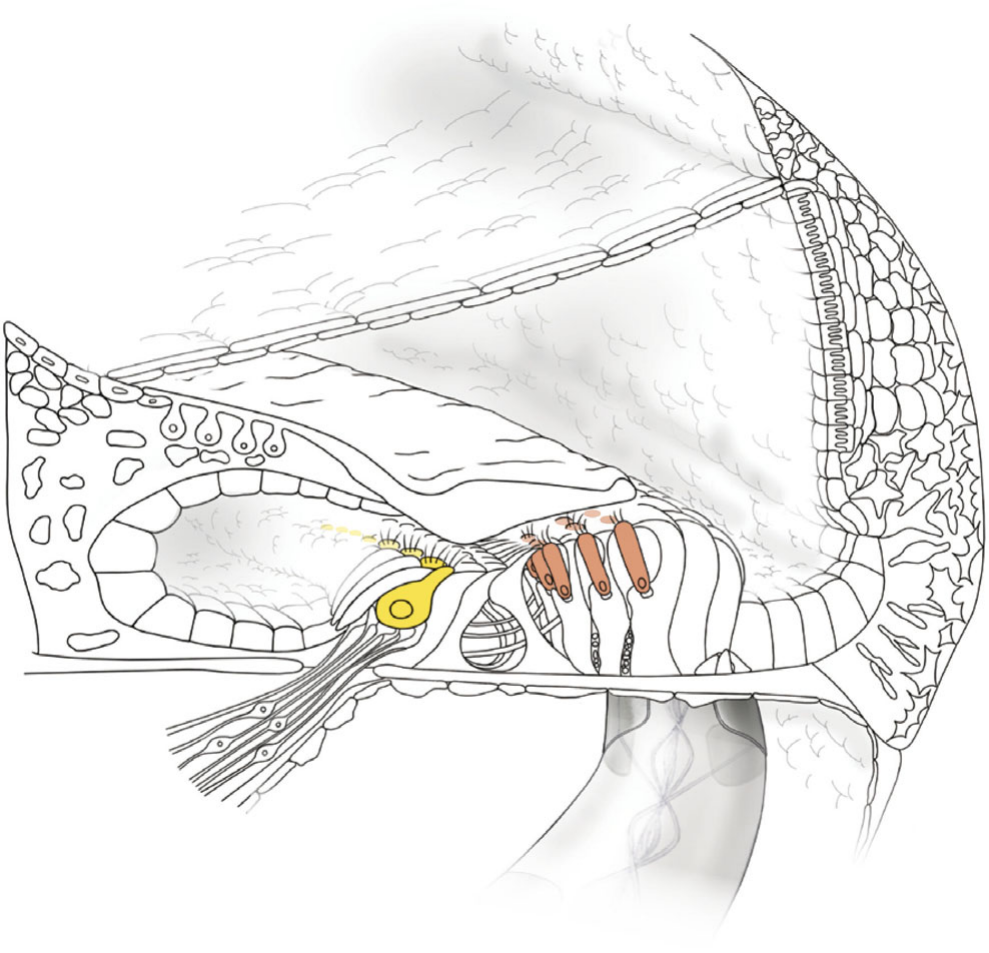
*LOXHD1* is predominantly expressed in inner and outer hair cells (Nishio et al., 2017).

*LOXHD1*, a known cause of DFNB77, is the gene for the protein Lipoxygenase Homology Domains 1, comprised entirely of 15 PLAT (polycystin‐1, lipoxygenease, alpha‐toxin) domains (Müller and Grillet, 2010). Murine *Loxhd1* proteins are expressed in hair cells, and are found in the cell membrane of the stereocilia, which is important for maintaining normal hair cell function (Grillet et al., 2009) (Fig. 35). Deterioration of hair cells in the cochlea, quickly lost after birth, is thought to be the cause of hearing loss in *Loxhd1* mutant mice.

**Figure 36:**
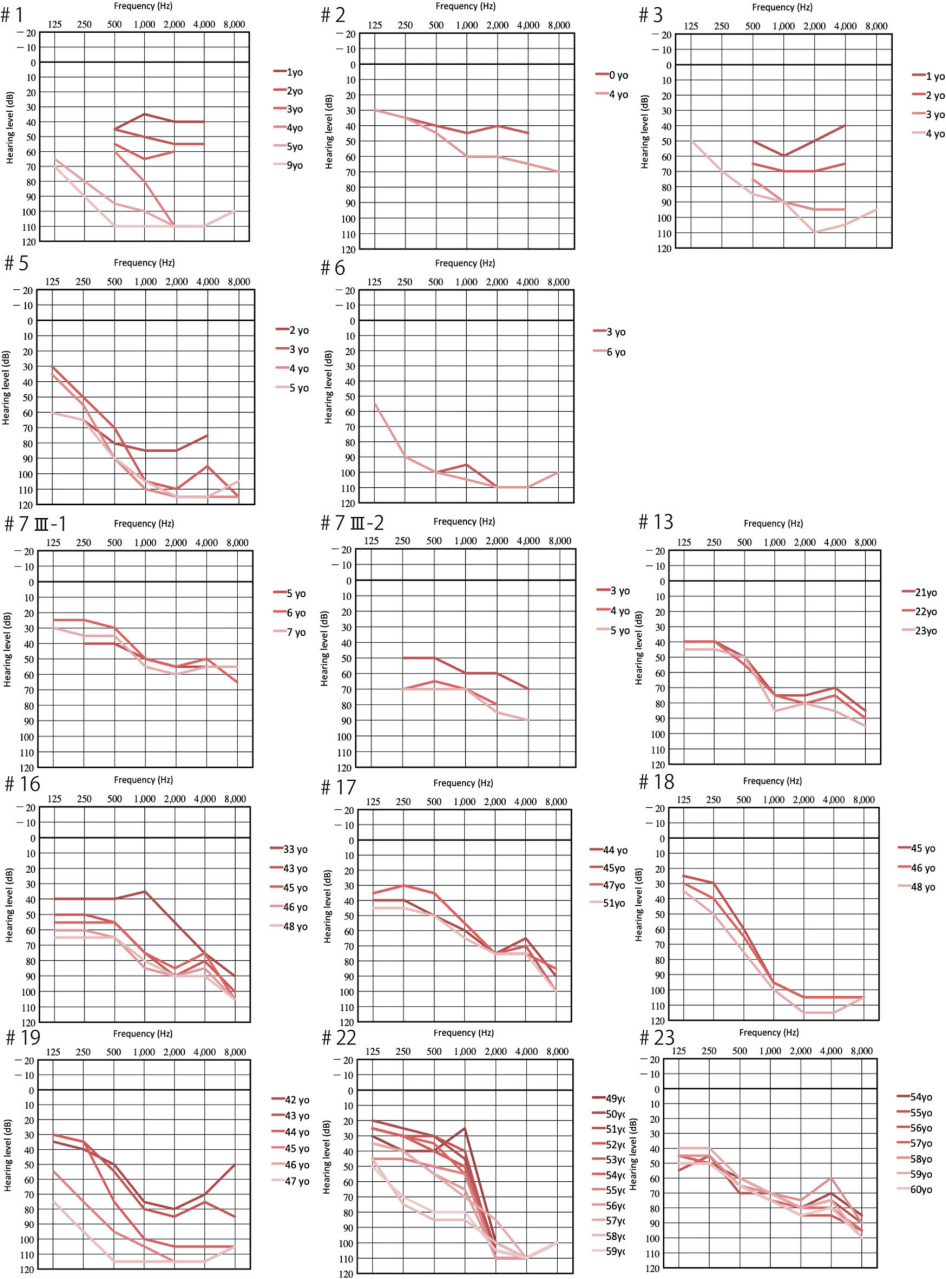
Serial audiograms of eight individuals with *LOXHD1* variations, indicating that hearing loss is high‐frequency involved and progressive. Darker colored lines: audiograms at younger ages, lighter colored lines: audiograms at older ages (Maekawa et al., 2019).

**Figure 37:**
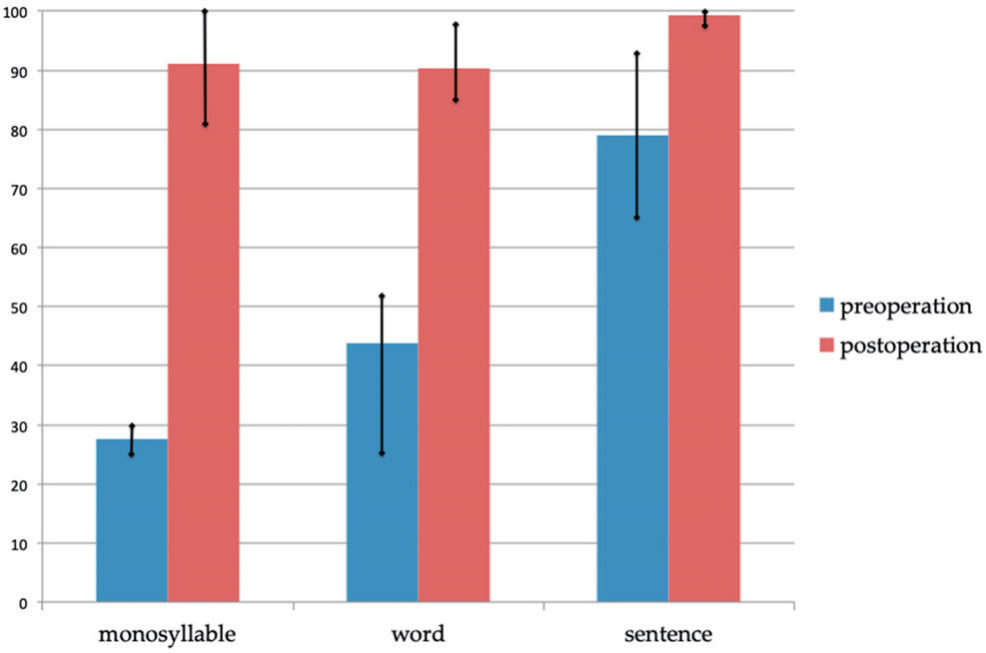
Mean scores of the Japanese monosyllable, word, and sentence tests pre‐CI and six months post‐CI. The scores pre‐CI are with hearing aids (Maekawa et al., 2019).

A series of studies have shown that patients with *LOXHD1* mutations show progressive hearing loss, leading to profound‐to‐severe non‐syndromic hearing loss (Mori et al., 2015; Maekawa et al., 2019). By examining a large cohort of hearing loss patients (n = 8,074), we have recently reported 28 patients with *LOXHD1* mutations and clarified the clinical features (Maekawa et al., 2019). Concerning the audiogram configurations, most of the patients had high‐frequency hearing loss (Fig. 36). The most notable characteristic is the progressiveness of hearing loss (Fig. 36). More than half of the individuals (15/28; 53.6%) were aware of the progression of hearing loss at the time of their genetic testing and 28.55% (8/28) of *LOXHD1* patients have received CI. All CI patients, whose clinical data were available, showed a favorable outcome (Fig. 37) (Maekawa et al., 2019). Therefore, patients with this gene mutation are good candidates for CI/EAS.

### Case: DFNB77 Caused by *LOXHD1* Mutations

#### *Congenital high‐frequency involved hearing loss indicative for EAS*

**Figure 38:**
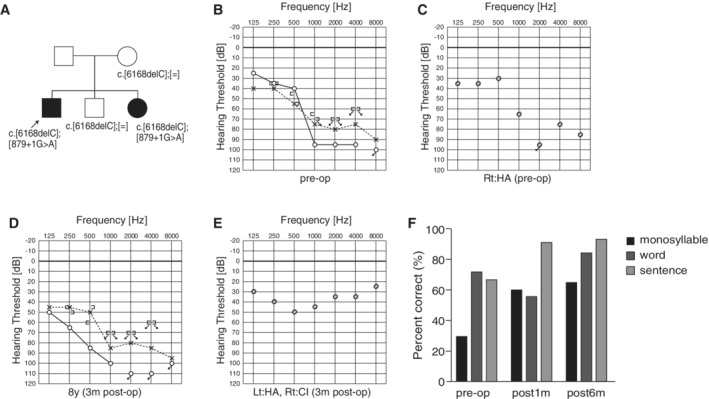
The EAS patient with *LOXHD1* mutations. **A**: The patient has compound heterozygous *LOXHD1* mutations, and his sister with hearing loss also carried the same compound heterozygous mutation. **B**: Preoperative audiogram. **C**: Preoperative hearing threshold with hearing aid. **D**: Postoperative audiogram (three months after EAS). **E**: Hearing threshold with EAS. **F**: Speech perception score of this patient showed good outcome.

He was diagnosed with hearing loss at age 3 and started wearing hearing aids (Maekawa et al., 2019). Evaluated by serial audiograms, it was determined that he had progressive hearing loss. At age 20, he visited our hospital due to insufficient amplification by hearing aids. Genetic testing identified *LOXHD1* compound heterozygous mutations (c.[6168delC];[879+1G>A]) (Fig. 38A). His sister had the same mutations, and had received CI. Considering residual hearing in the lower frequencies and possible future deterioration of hearing as a natural course, EAS using a long electrode (MEDEL Synchrony/FLEX28 electrode) was chosen for the right ear. Residual hearing for sufficient acoustic amplification was preserved (Fig. 38D). His speech discrimination score was improved from 30% to 65% (Fig. 38F).

#### *COCH*

**Figure 39:**
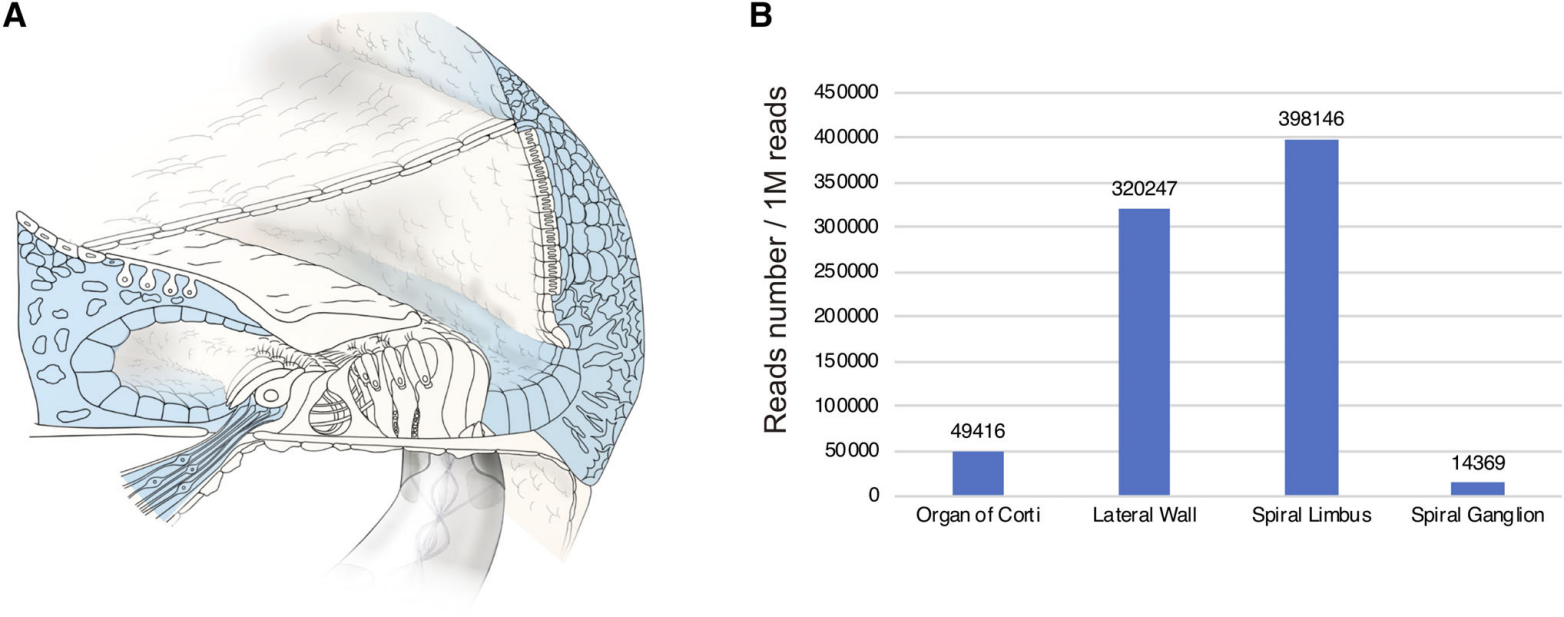
**A**: *COCH* is abundantly expressed in the cochlea, especially in inner and outer sulcus cells, the spiral ligament, and the spiral limbus. **B**: *Coch* gene expression in the mouse cochlea and spiral ganglion (modified from Nishio et al., 2017).

*COCH* encodes extracellular protein “cochlin” that consists of a single peptide, a late gestation lung protein Lgl1 (LCCL) domain, and two von Willebrand factor A (vWFA) domains (Robertson, 1997). Cochlin is highly expressed in the cochlea, and while the protein function has not been well characterized, it is thought to play an important role in the function of the inner ear (Fig. 39). *COCH* mutations are reported to cause autosomal dominant sensorineural hearing loss with vestibular dysfunction (DFNA9) (Robertson et al., 1998). Histological studies investigating patients with *COCH* mutations found acidophilic deposits in the vestibular endorgans and cochlea, and cell loss, as well as that of cochlear dendrites (Khetarpal et al., 1991; Bom et al., 1999).

With regard to the effectiveness of CI, Nagy et al. reported that CI had no benefit in patients with *COCH* mutations (Nagy et al., 2004), while other reports, including our own, have described improved results after CI (Street et al., 2005; Vermeire et al., 2006; Tsukada et al., 2015). A previous temporal bone study reported that spiral ganglions were able to survive even though there was extensive degradation of cochlear dendrites (Khetarpal et al., 1991), indicating that the remaining spiral ganglion cells can be stimulated by CI, resulting in an improved performance after CI in patients with *COCH* mutations.

### Case: DFNA9 Caused by a *COCH* Mutation

#### *Adult‐onset progressive hearing loss with vestibular dysfunction*

**Figure 40:**
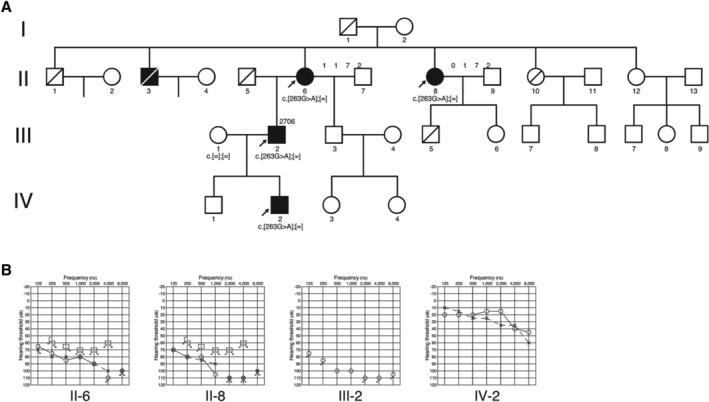
**A**: Pedigree and **B**: audiograms of the family with p.G88E mutations in the *COCH* gene (Tsukada et al., 2015).

**Figure 41:**
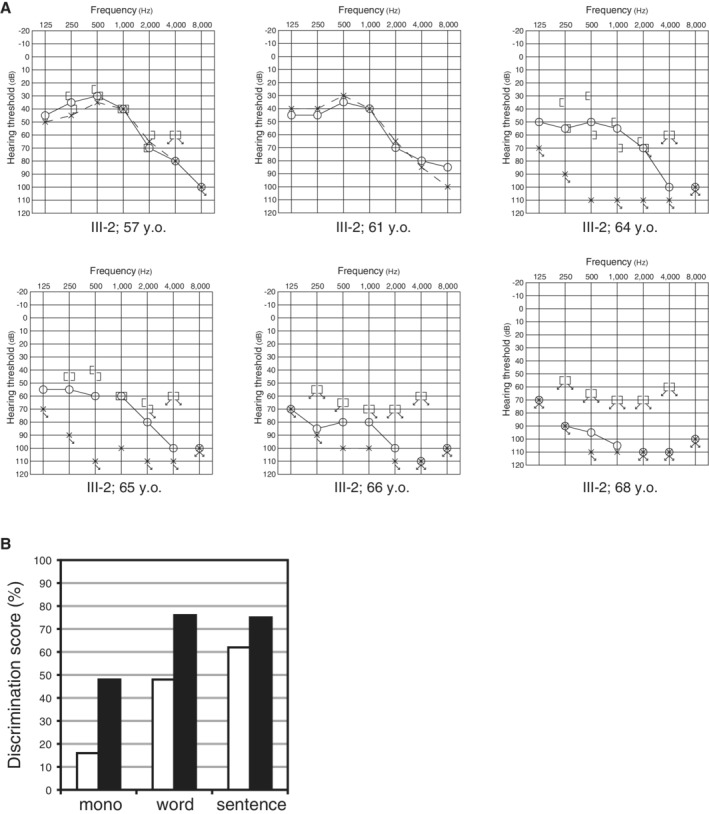
**A**: Acute deterioration of hearing in proband III‐2 was found between 64 and 68 years old. **B**: The speech perception scores were improved after CI (monosyllables, words, and sentences). White bars: pre‐CI, black bars: post‐CI (Tsukada et al., 2015).

The proband was a 70‐year‐old man (III‐2) (Fig. 40) (Tsukada et al., 2015). In his early 50s he experienced hearing loss for the first time, which was not accompanied with tinnitus or vertigo. He first visited our hospital at age 57. High‐frequency hearing loss was measured by pure‐tone audiograms. Speech discrimination scores for the right and left ear were 80% and 90%, respectively. The patient had a rapid progression in hearing loss starting at age 64. At the age of 65, he was found to have bilateral deafness (Fig. 41A). Tests revealed a 16% speech discrimination score (Fig. 41B). At the age of 68, implantation of the right ear with a MED‐EL PULSAR^/^FLEXSOFT electrode was performed. CI improved his speech perception scores to 76% and 75%, compared to 43% and 63% preoperatively with hearing aids, respectively (Fig. 41B).

## CONCLUSION

The above results suggest that the genes presented may be involved in hearing loss in CI patients. As patients with these mutations showed relatively good auditory performance after receiving CI or EAS, genetic background could be added as one of the factors useful in predicting performance after implantation. Also genetic testing is helpful in predicting future hearing levels; therefore, genetic diagnosis could facilitate decision making for early intervention. Furthermore, genetic testing is potentially useful for evaluating residual hearing, estimating progression, and successful hearing preservation making it valuable in the selection of candidates and electrodes for CI or EAS.

## CONFLICT OF INTEREST

The authors declare no conflicts of interest. The funders had no role in the design of the study; in the collection, analyses, or interpretation of the data; in the writing of the manuscript, or in the decision to publish the results.
